# Snapshot Europe 2021–2022: The first public dataset from a Europe‐wide camera‐trap survey of terrestrial vertebrates

**DOI:** 10.1002/ecy.70516

**Published:** 2026-11-05

**Authors:** Fabiola Iannarilli, Brigitta Keeves‐von Wolff, Martin Wikelski, Marco Heurich, Roland Kays, William J. McShea, Michael V. Cove, Alexandros Α. Karamanlidis, Lambros Krambokoukis, Andrea Gazzola, Teodora Sin, Barbara Pregler, Owain Barton, Graeme Shannon, Sina Greiner, Rudolf Reiner, Ferran Sayol, Marc Vilella, José Vicente López‐Bao, Patricia Mateo‐Tomás, Raffael Kratzer, Paul Griesberger, Klaus Hackländer, Elisabeth Wesely, Sebastian Brackhane, Andreas Daim, Felicitas Oehler, Sophie Nöbauer, Frederik Sachser, Janosch Arnold, Miroslav Kutal, Ana Luísa Barros, Margarida Santos‐Reis, Viorel D. Popescu, Valentin Moser, Lorenz Achtnich, Mareike Kortmann, Magne Neby, Lorenzo Frangini, Camilo Carneiro, Ilaria Greco, Francesco Rovero, Marco Salvatori, Alessandro Massolo, Orlando Tomassini, Ninon F. V. Meyer, Anne Peters, Lukas G. Scholz, Thomas Kudernatsch, Mihael Janječić, Nikica Šprem, Zbigniew Borowski, Benedikt A. Gehr, Claude A. Andrist, Javier Calzada, Santiago Gutiérrez‐Zapata, Simone Santoro, Lucrezia Lorenzetti, Valerio Donini, Elisa Iacona, Sandro Bertolino, Elizabeth F. R. Preston, Kristy A. Adaway, Matthew C. Morgan, F. Blake Morton, Koko M. Sutter, Dylan Thompson‐Jones, Laura Juvany, Tim R. Hofmeester, Sönke Twietmeyer, Giacomo Cremonesi, Francesco Bisi, Elena Bužan, Luka Duniš, Emiliano Manzo, Nadia Cappai, Ruben Iosif, Barbara Promberger‐Fuerpass, Adam F. Smith, Colin Brock, Simone Ciuti, Lee Oliver, Scott M. Appleby, Gunnar Jansson, Alisa Klamm, Axel Ballmann, József Lanszki, Maximilian L. Allen, Andrea De Giovanni, Nadège C. Bonnot, Anders Mårell, Andrius Pašukonis, Izabela A. Wierzbowska, Lara Indra, Barbara Franzetti, Pietro Pontiggia, Alper Ertürk, Anil Soyumert, Guna Bagrade, Aivars Ornicāns, Jānis Ozoliņš, Alda Stepanova, Marc Fusellas, Andrej Gonev, Dime Melovski, Aleksandar Pavlov, Marcin Churski, Monika M. Gehrke, Katharina Kasper, Anna J. Kirsch, Dries P. J. Kuijper, Elise Say‐Sallaz, Paulina Anna Szafrańska, Jose Vila‐López, Šárka Frýbová, Joana Colomer Rosell, Stanisław Pagacz, Julia Witczuk, Anamarija Žagar, Valentin Specht, Sam Vereecken, Laura Braunholtz, Aileen C. Mill, Bethany E. Patterson, Marion Pfeifer, Amir Arnon, Niko Boone, Jim Casaer, Lynn Pallemaerts, Matthias‐Claudio Loretto, J. David Blount, Çağan H. Şekercioğlu, Josip Kusak, Emrah Coban, Thomas Cansse, Annika M. Felton, Ursula Sterrer, Michał Wagner, Emilia Zaborowska, Salvador Salvador, Hannah D. Broeckx, Miha Krofel, Eszter Nagy, Mark Bowler, Triin Kaasiku, David G. Tosh, Alekss Vecvanags, Manuel E. Gegundez Arias, Katarzyna Szyc, Hendrik Edelhoff, Wibke Peters, Niko Balkenhol, Kathrin Mayer, Johannes Signer, André Fetzer, Johannes Lang, Johann D. Lanz, Teresa F. Nava, Federico Ossi, Francesca Cagnacci

**Affiliations:** ^1^ Department of Migration Max Planck Institute of Animal Behavior Radolfzell Germany; ^2^ Department of Biology University of Konstanz Constance Germany; ^3^ Bavarian Forest National Park Grafenau Germany; ^4^ University of Freiburg Freiburg Germany; ^5^ University of Inland Norway Elverum Norway; ^6^ North Carolina Museum of Natural Sciences Raleigh North Carolina USA; ^7^ North Carolina State University Raleigh North Carolina USA; ^8^ Smithsonian's National Zoo and Conservation Biology Institute Washington DC USA; ^9^ ARCTUROS – Civil Society for the Protection and Management of Wildlife and the Natural Environment Aetos, Florina Greece; ^10^ Association for the Conservation of Biological Diversity Focșani Romania; ^11^ Centre for Environmental Research and Impact Studies University of Bucharest Bucharest Romania; ^12^ Babia Gora National Park Zawoja Poland; ^13^ Bangor University Bangor UK; ^14^ University of Alberta Edmonton Alberta Canada; ^15^ Berchtesgaden National Park Berchtesgaden Germany; ^16^ BETA Technological Centre – University of Vic – Central University of Catalonia (BETA‐UVIC‐UCC) Vic, Barcelona Spain; ^17^ Bibio Research Group, Natural Sciences Museum of Granolllers Granollers Spain; ^18^ Biodiversity Research Institute (CSIC–University of Oviedo–Principality of Asturias) Mieres Spain; ^19^ Black Forest National Park Seebach Germany; ^20^ BOKU University, Institute of Wildlife Biology and Game Management, Department of Ecosystem Management Climate and Biodiversity Vienna Austria; ^21^ Deutsche Wildtier Stiftung Hamburg Germany; ^22^ Wildlife Research Unit Baden‐Württemberg, LAZBW Aulendorf Germany; ^23^ Carnivore Conservation Programme, Friends of the Earth, Czech Republic Olomouc Czech Republic; ^24^ Department of Forest Ecology Mendel University in Brno Brno Czech Republic; ^25^ Centre for Ecology, Evolution and Environmental Changes, Faculdade de Ciências Universidade de Lisboa Lisbon Portugal; ^26^ Global Change and Sustainability Institute, Faculdade de Ciências Universidade de Lisboa, Campo Grande Lisbon Portugal; ^27^ Columbia University New York New York USA; ^28^ University of Bucharest, Romania Bucharest Romania; ^29^ Community Ecology, Swiss Federal Institute for Forest, Snow and Landscape Research WSL Birmensdorf Switzerland; ^30^ Department of Aquatic Ecology, Eawag: Swiss Federal Institute of Aquatic Science and Technology Dübendorf Switzerland; ^31^ Projectfelis.org Kehrsatz Switzerland; ^32^ Conservation Biology and Forest Ecology Julius‐Maximilians‐University Würzburg Würzburg Germany; ^33^ Department of Agricultural Sciences University of Inland Elverum Norway; ^34^ Department of Agricultural, Food, Environmental and Animal Sciences University of Udine Udine Italy; ^35^ Department of Biology & CESAM University of Aveiro, Campus de Santiago Aveiro Portugal; ^36^ South Iceland Research Centre University of Iceland Laugarvatn Iceland; ^37^ Department of Biology University of Florence Florence Italy; ^38^ MUSE – Science Museum Trento Italy; ^39^ Department of Biology University of Pisa Pisa Italy; ^40^ Department of Biology University of Toronto Mississauga Ontario Canada; ^41^ Wildlife Ecology and Management University of Freiburg Freiburg Germany; ^42^ Department of National Park Monitoring and Animal Management Bavarian Forest National Park Grafenau Germany; ^43^ Department of Conservation and Biodiversity Bavarian State Institute of Forestry Freising Germany; ^44^ Department of Fisheries, Apiculture, Wildlife Management and Special Zoology, Faculty of Agriculture University of Zagreb Zagreb Croatia; ^45^ Department of Forest Ecology Forest Research Institute Raszyn Poland; ^46^ Department of Evolutionary Biology and Environmental Sciences University of Zurich Zurich Switzerland; ^47^ Wildtier Schweiz University of Zurich Zurich Switzerland; ^48^ Department of Integrated Sciences & Center for Advanced Studies in Physics, Mathematics, and Computing, Faculty of Experimental Sciences University of Huelva Huelva Spain; ^49^ Department of Integrated Sciences, Faculty of Experimental Sciences University of Huelva Huelva Spain; ^50^ Department of Natural Sciences and Environmental Health University of South‐Eastern Norway Borre Norway; ^51^ Department of Life Sciences University of Siena Siena Italy; ^52^ Stelvio National Park Bormio Italy; ^53^ Department of Life Sciences and Systems Biology University of Turin Turin Italy; ^54^ Department of Pathobiology and Population Sciences Royal Veterinary College London UK; ^55^ Department of Psychology University of Hull Hull UK; ^56^ Department of Wildlife, Fish, and Environmental Studies Swedish University of Agricultural Sciences Umeå Sweden; ^57^ Eifel National Park Schleiden Germany; ^58^ Environment Analysis and Management Unit – Guido Tosi Research Group, Department of Theoretical and Applied Sciences University of Insubria Varese Italy; ^59^ Faculty of Environmental Protection Velenje Slovenia; ^60^ University of Primorska, Faculty of Mathematics, Natural Sciences and Information Technologies Koper Slovenia; ^61^ Fondazione Ethoikos, Convento dell'Osservanza S.N.C. Radicondoli, Siena Italy; ^62^ Foreste Casentinesi National Park Pratovecchio Italy; ^63^ Foundation Conservation Carpathia Brașov Romania; ^64^ Frankfurt Zoological Society Frankfurt Germany; ^65^ Laboratory of Wildlife Ecology and Behaviour, SBES University College Dublin Dublin Ireland; ^66^ Game & Wildlife Conservation Trust London UK; ^67^ Georg‐August‐Universität Göttingen Göttingen Germany; ^68^ Grimsö Wildlife Research Station, Department of Ecology Swedish University of Agricultural Sciences Uppsala Sweden; ^69^ Department of Conservation and Research Hainich National Park Bad Langensalza Germany; ^70^ Harz National Park Wernigerode Germany; ^71^ HUN‐REN Balaton Limnological Research Institute Tihany Hungary; ^72^ Illinois Natural History Survey, Prairie Research Institute University of Illinois Champaign Illinois USA; ^73^ Independent Researcher Bolzano Italy; ^74^ INRAE, EFNO, Domaine des Barres Nogent‐sur‐Vernisson France; ^75^ Université de Toulouse, INRAE, CEFS Castanet‐Tolosan France; ^76^ Institute of Biosciences, Life Sciences Center Vilnius University Vilnius Lithuania; ^77^ Institute of Environmental Sciences, Faculty of Biology Jagiellonian University Kraków Poland; ^78^ Institute of Forensic Medicine University of Bern Bern Switzerland; ^79^ Italian Institute for Environmental Protection and Research Rome Italy; ^80^ Kastamonu University Arac Rafet Vergili Vocational School of Higher Education, Hunting and Wildlife Programme Arac Kastamonu Türkiye; ^81^ Latvian State Forest Research Institute “Silava” Salaspils Latvia; ^82^ Limnos Banyoles Spain; ^83^ Macedonian Ecological Society Skopje North Macedonia; ^84^ Mammal Research Institute, Polish Academy of Science Białowieża Poland; ^85^ Mendel University in Brno Brno Czech Republic; ^86^ Minuartia Barcelona Spain; ^87^ Department of Evolutionary Biology, Ecology and Environmental Sciences Universitat de Barcelona Barcelona Spain; ^88^ Museum and Institute of Zoology Polish Academy of Sciences Warsaw Poland; ^89^ National Institute of Biology NIB Ljubljana Slovenia; ^90^ Nationalpark Donau‐Auen Orth an der Donau Austria; ^91^ Natuurpunt Scheldeland‐kern Berlare Berlare Belgium; ^92^ Newcastle University Newcastle upon Tyne UK; ^93^ Ramat Hanadiv Nature Park Zikhron Ya'akov Israel; ^94^ Research Institute for Nature and Forest (INBO) Brussels Belgium; ^95^ Research Institute of Wildlife Ecology, Department of Interdisciplinary Life Sciences University of Veterinary Medicine Vienna Austria; ^96^ School of Biological Sciences, University of Utah Salt Lake City Utah USA; ^97^ Department of Molecular Biology and Genetics Koç University Istanbul Turkey; ^98^ KuzeyDoğa Society Kars Turkey; ^99^ Veterinary Biology Unit, Veterinary Faculty Zagreb Zagreb Croatia; ^100^ School of Life and Environmental Sciences, Faculty of Science, Engineering and Built Environment Deakin University Burwood Victoria Australia; ^101^ Southern Swedish Forest Research Centre Swedish University of Agricultural Sciences Uppsala Sweden; ^102^ Swiss National Park Zernez Switzerland; ^103^ Taxus SI Sp. z o.o. Warszawa Poland; ^104^ Universitat de Girona Girona Spain; ^105^ University of Amsterdam Amsterdam The Netherlands; ^106^ Biotechnical Faculty University of Ljubljana Ljubljana Slovenia; ^107^ Institute of Wildlife Biology and Wildlife Management University of Sopron Sopron Hungary; ^108^ University of Suffolk Ipswich UK; ^109^ University of Tartu Tartu Estonia; ^110^ University of Ulster Coleraine UK; ^111^ Vides Risinājumu Institūts (Institute for Environmental Solutions) Priekuļi Latvia; ^112^ Vision, Prediction, Optimisation and Control Systems Department, Science and Technology Research Centre University of Huelva Huelva Spain; ^113^ Warsaw University of Life Sciences Warsaw Poland; ^114^ Wildlife Biology and Management Research Unit Bavarian State Institute of Forestry Freising Germany; ^115^ Wildlife Sciences, Faculty of Forest Sciences and Forest Ecology University of Goettingen Goettingen Germany; ^116^ Working Group for Wildlife Research at the Clinic for Birds, Reptiles, Amphibians and Fish Justus Liebig University Giessen Giessen Germany; ^117^ Animal Ecology Unit, Research and Innovation Centre, Fondazione Edmund Mach San Michele all'Adige Italy; ^118^ National Biodiversity Future Centre (NBFC) Palermo Italy

**Keywords:** annual surveys, biodiversity, biogeography, birds, camera traps, conservation, Europe, mammals, occupancy, species distributions, transnational monitoring

## Abstract

Managing and conserving species in the face of the current biodiversity crisis requires consistent information on the status of wild animal populations across large spatiotemporal scales. Budgetary constraints, differences in national legislation, and the challenges of coordinating logistics transnationally often limit survey efforts to spatial extents that rarely encompass the full distribution of many species, and temporal extents that rarely exceed a few years. These limitations are particularly relevant in regions like Europe, where multiple countries often share and manage populations of the same wild species across national borders and under different legislative frameworks. Cooperation and collaboration can help overcome some of these challenges. Here, we present data from the first 2 years of Snapshot Europe, a collaborative, coordinated, and standardized European‐wide mammal survey conducted by camera‐trap experts working across the continent. Between mid‐August and mid‐November of 2021 and 2022, more than 160 researchers simultaneously collected camera‐trap data at 1198 and 967 locations across 81 and 55 arrays and 22 and 23 countries, respectively, for a total of 90,230 camera‐trap days (49,033 and 41,197 in 2021 and 2022, respectively), with 91% of the data collected in September and October. Thirty (28%) out of 106 arrays across the 2 years were sampled both in 2021 and 2022, allowing for comparisons of estimates over time. All data were managed and processed in Wildlife Insights (https://www.wildlifeinsights.org/); each record was reviewed by at least one participant who confirmed or edited the initial classifications provided by the AI model integrated into the platform. The final dataset contains a total of 94,441 observations of 63 species of mammals and 5024 detections of 78 species of birds. These data capture a subsample of the European wildlife community and can be used to explore ecological questions related to drivers of distribution, abundance, and activity across the continent. We envision repeating this effort annually for the foreseeable future. Data for Snapshot Europe 2023, 2024, and 2025 are currently being revised and will be made publicly available soon. Collated, all the annual datasets will enable estimates of trends over time. Additionally, four sister initiatives, Snapshot USA (2019‐ongoing), Snapshot Japan (2023‐ongoing), Snapshot Brazil and Snapshot Chile (both started in 2025), annually collect data based on the same sampling protocol, enabling a new era of global‐scale comparisons. The images associated with the data accompanying this publication are available in Wildlife Insights. The data are available for reuse under CC BY‐SA 4.0 licensing. Please cite this data paper when reusing all or part of this dataset.

## CONFLICT OF INTEREST STATEMENT

The authors declare no conflicts of interest.

## Supporting information


Data S1.

## Data Availability

Data and metadata are available as Supporting Information in Data S1. The data are also available in Edmond—the Open Research Data Repository of the Max Planck Society at https://doi.org/10.17617/3.7YLWMU. The original images used to compile this dataset are available on Wildlife Insights (Snapshot Europe 2021, http://n2t.net/ark:/63614/w12003388; Snapshot Europe 2022, http://n2t.net/ark:/63614/w12004446).

