# Supplementary material for: Snapshot Europe 2021–2022: The first public dataset from a Europe‐wide camera‐trap survey of terrestrial vertebrates
Source: Ecology. 2026 Nov 5;107(11):e70516. doi: 10.1002/ecy.70516 (PMC13740659; doi:10.1002/ecy.70516)
Supplement: Supplementary file 1 [file ECY-107-e70516-s001.zip › Metadata_S1.pdf]

Data Paper published in *Ecology*, an Ecological Society of America journal

Metadata S1

Snapshot Europe 2021–2022: The first public dataset from a Europe-wide camera-trap survey of terrestrial vertebrates

Fabiola Iannarilli, Brigitta Keeves-von Wolff, Martin Wikelski, Marco Heurich, Roland Kays, William J. McShea, Michael V. Cove, Alexandros A. Karamanlidis, Lambros Krambokoukis, Andrea Gazzola, Teodora Sin, Barbara Pregler, Owain Barton, Graeme Shannon, Sina Greiner, Rudolf Reiner, Ferran Sayol, Marc Vilella, José Vicente López-Bao, Patricia Mateo-Tomás, Raffael Kratzer, Paul Griesberger, Klaus Hackländer, Elisabeth Wesely, Sebastian Brackhane, Andreas Daim, Felicitas Oehler, Sophie Nöbauer, Frederik Sachser, Janosch Arnold, Miroslav Kutal, Ana Luísa Barros, Margarida Santos-Reis, Viorel D. Popescu, Valentin Moser, Lorenz Achtnich, Mareike Kortmann, Magne Neby, Lorenzo Frangini, Camilo Carneiro, Ilaria Greco, Francesco Rovero, Marco Salvatori, Alessandro Massolo, Orlando Tomassini, Ninon F.V. Meyer, Anne Peters, Lukas G. Scholz, Thomas Kudernatsch, Mihael Janječić, Nikica Šprem, Zbigniew Borowski, Benedikt A. Gehr, Claude A. Andrist, Javier Calzada, Santiago Gutiérrez-Zapata, Simone Santoro, Lucrezia Lorenzetti, Valerio Donini, Elisa Iacona, Sandro Bertolino, Elizabeth F.R. Preston, Kristy A. Adaway, Matthew C. Morgan, F. Blake Morton, Koko M. Sutter, Dylan Thompson-Jones, Laura Juvany, Tim R. Hofmeester, Sönke Twietmeyer, Giacomo Cremonesi, Francesco Bisi, Elena Bužan, Luka Duniš, Emiliano Manzo, Nadia Cappai, Ruben Iosif, Barbara Promberger-Fuerpass, Adam F. Smith, Colin Brock, Simone Ciuti, Lee Oliver, Scott M. Appleby, Gunnar Jansson, Alisa Klamm, Axel Ballmann, József Lanszki, Maximilian L. Allen, Andrea De Giovanni, Nadège C. Bonnot, Anders Mårell, Andrius Pašukonis, Izabela A. Wierzbowska, Lara Indra, Barbara Franzetti, Pietro Pontiggia, Alper Ertürk, Anil Soyumert, Guna Bagrade, Aivars Ornicāns, Jānis Ozoliņš, Alda Stepanova, Marc Fusellas, Andrej Gonev, Dime Melovski, Aleksandar Pavlov, Marcin Churski, Monika M. Gehrke, Katharina Kasper, Anna J. Kirsch, Dries P.J. Kuijper, Elise Say-Sallaz, Paulina Anna Szafrańska, Jose Vila-López, Šárka Frýbová, Joana Colomer Rosell, Stanisław Pagacz, Julia Witczuk, Anamarija Žagar, Valentin Specht, Sam Vereecken, Laura Braunholtz, Aileen C. Mill, Bethany E. Patterson, Marion Pfeifer, Amir Arnon, Niko Boone, Jim Casaer, Lynn Pallemaerts, Matthias-Claudio Loretto, J. David Blount, Çağan H. Şekercioğlu, Josip Kusak, Emrah Coban, Thomas Cansse, Annika M. Felton, Ursula Sterrer, Michał Wagner, Emilia Zaborowska, Salvador Salvador, Hannah D. Broeckx, Miha Krofel, Eszter Nagy, Mark Bowler, Triin Kaasiku, David G. Tosh, Alekss Vecvanags, Manuel E. Gegundez Arias, Katarzyna Szyc, Hendrik Edelhoff, Wibke Peters, Niko Balkenhol, Kathrin Mayer, Johannes Signer, André Fetzer, Johannes Lang, Johann D. Lanz, Teresa F. Nava, Federico Ossi, Francesca Cagnacci

Class I. Data Set Descriptors

A. Data set identity: Camera-trap data of terrestrial mammals and birds from the first two years of Snapshot Europe, a coordinated, expert-based camera-trap survey of areas across 22 (2021) and 23 (2022) European countries.

B. Data set identification codes:

Snapshot_Europe_2021_2022_cameras.csv;
Snapshot_Europe_2021_2022_deployments.csv;
Snapshot_Europe_2021_2022_projects.csv;
Snapshot_Europe_2021_2022_sequences.csv;
Snapshot_Europe_2021_2022_images.csv

C. Data set description

1. Originators:

- Fabiola Iannarilli, Max Planck Institute of Animal Behavior, Germany;
- Brigitta Keeves-von Wolff, Max Planck Institute of Animal Behavior, Germany;
- Martin Wikelski, Max Planck Institute of Animal Behavior, Germany and Department of Biology, University of Konstanz, Konstanz, Germany;
- Marco Heurich, Bavarian Forest National Park, Germany, University of Freiburg, Germany, and University of Inland Norway, Norway
- Roland Kays, North Carolina Museum of Natural Sciences, USA and North Carolina State University, USA
- William J. McShea, Smithsonian's National Zoo and Conservation Biology Institute, USA
- Michael V. Cove, North Carolina Museum of Natural Sciences, USA
- Francesca Cagnacci, Fondazione Edmund Mach, Italy and National Biodiversity Future Centre (NBFC), Palermo, Italy

2. Abstract: Managing and conserving species in the face of the current biodiversity crisis requires consistent information on the status of wild animal populations across large spatio-temporal scales. Budgetary constraints, differences in national legislation, and the challenges of coordinating logistics transnationally often limit survey efforts to spatial extents that rarely encompass the full distribution of many species, and temporal extents that rarely exceed a few years. These limitations are particularly relevant in regions like Europe, where multiple countries often share and manage populations of the same wild species across national borders and under different legislative frameworks. Cooperation and collaboration can help overcome some of these challenges. Here, we present data from the first two years of Snapshot Europe, a collaborative, coordinated, and standardised European-wide mammal survey conducted by camera-trap experts working across the continent. Between mid-August and mid-November of 2021 and 2022, more than 160 researchers simultaneously collected camera-trap data at 1,198 and 967 locations across 81 and 55 arrays and 22 and 23 countries, respectively, for a total of 90,230 camera-trap days (49,033 and 41,197 in 2021 and 2022, respectively), with 91% of the data collected in September and October. Thirty (28%) out of 106 arrays across the two years were sampled both in 2021 and 2022, allowing for comparisons of estimates over time. All data were

managed and processed in Wildlife Insights (https://www.wildlifeinsights.org/); each record was reviewed by at least one participant who confirmed or edited the initial classifications provided by the AI model integrated into the platform. The final dataset contains a total of 94,441 observations of 63 species of mammals and 5,024 detections of 78 species of birds. These data capture a sub-sample of the European wildlife community and can be used to explore ecological questions related to drivers of distribution, abundance and activity across the continent. We envision repeating this effort annually for the foreseeable future. Data for Snapshot Europe 2023, 2024, and 2025 are currently being revised and will be made publicly available soon. Collated, all the annual datasets will enable estimates of trends over time. Additionally, four sister initiatives, Snapshot USA (2019-ongoing), Snapshot Japan (2023-ongoing), Snapshot Brazil and Snapshot Chile (both started in 2025) annually collect data based on the same sampling protocol, enabling a new era of global-scale comparisons. The images associated with the data accompanying this publication are available in Wildlife Insights. There are no copyright restrictions on the use of these data; however, we ask interested users to please cite this publication when using all or part of this dataset.

D. Key words/phrases: annual surveys, biodiversity, biogeography, birds, camera traps, conservation, Europe, mammals, occupancy, species distributions, transnational monitoring.

Class II. Research origin descriptors

A. Overall project description:

1. Identity: Snapshot Europe 2021–2022: The first public dataset from a Europe-wide camera-trap survey of terrestrial vertebrates

2. Originators: see Class I section C1.

3. Period of study:

- Snapshot Europe 2021: August 18, 2021 - November 15, 2021;
- Snapshot Europe 2022: August 15, 2022 - November 15, 2022;

4. Objectives: The Snapshot Europe initiative aims to create a continental-wide database of public wildlife data by fostering collaboration among researchers working on wildlife conservation and management. Snapshot Europe's objectives are twofold:

a. collecting camera-trap data across Europe on an annual basis and providing information to explore trends and ecological patterns at the European scale to inform the conservation and management of wild species;
b. leveraging the compelling imagery collected to increase awareness of wildlife issues among the public through outreach and educational activities and opportunities.

By making this data publicly available, Snapshot Europe aims to provide the ecological community information to assess and test hypotheses related to the abundance, distribution, and behavior of the species surveyed.

5. Abstract: Snapshot Europe 2021–2022 is the first publicly available dataset from standardised, coordinated, and collaborative surveys of terrestrial mammals and birds across Europe. The dataset includes camera-trap observations collected by expert researchers at 1,198 and 967 sites across 22 and 23 countries in 2021 and 2022, respectively (Fig. 1). Camera sites were clustered in 81 and 55 arrays (i.e. subprojects), respectively, with the average number of sites per array equal to 16 +/- 11 (mean +/- standard deviation; range: [2-62]) and the average number of camera-trap days equal to 663 +/- 501 (range: [80-3,005]; Table 1; Fig. 2). Locations of the sites within each array were chosen by the collaborator team managing that array, with sites spaced more than 200 m apart, but with each site less than 5 km from at least another. The areas sampled were representative of all the major ecoregions present in the European continent (Fig. 1) and included forests, wetlands, shrubby areas, and agricultural and urban landscapes. Data were organised in sequences (i.e. events), each consisting of all the images collected at a certain location less than 1 minute apart from each other. This 1-minute threshold was chosen to share observations at a fine temporal scale while still addressing most of the autocorrelation in the records (Kays & Parsons, 2014; Parsons et al., 2016; Yasuda, 2004). Using sequences also allowed us to gain more robust counts of animals in groups. All the collected observations of mammals and birds were included in the dataset. When possible (e.g., clear pictures, well-visible individuals), all observations of mammals and birds were reported at the species level. Smaller species (e.g. < 300 g) or species difficult to distinguish in images (e.g. *Apodemus* spp.) were reported at the lowest taxonomical level possible. This resulted in 94,441 sequences of 63 species of mammals and 5,024 sequences of 78 species of birds resolved at the species level (Table 2; Fig. 3); an additional 1367 sequences of mammals and 180 sequences of birds were resolved at the genus level. Red deer (*Cervus elaphus*), followed closely by roe deer (*Capreolus capreolus*), was the species most frequently detected. Other species frequently detected included wild boar (*Sus scrofa*), red fox (*Vulpes vulpes*), and European rabbit (*Oryctolagus cuniculus*). These species match the list of expected species based on the protocol adopted (unbaited cameras ~50 cm above and parallel to the ground), which is tailored towards medium to large-bodied terrestrial, ground-dwelling vertebrates (> 300g). The dataset also contains records of humans (including records of camera trappers engaged in the deployment, management, and retrieval of the cameras; 6,647 sequences in 2021, 7,767 in 2022, summarized after excluding research activities) and domestic animals (2,990 in 2021, 4,346 in 2022; Table 2).

This dataset will enable researchers to explore large-scale patterns in the distribution, abundance and activity of the species targeted. It will also provide insights into the effects of humans and domestic animal disturbance on wildlife. Across-year comparisons between these first two and future years (data from 2023, 2024, and 2025 are currently being processed) will also be possible thanks to the regularity in data collection, always centred on September - October (Fig. 2).

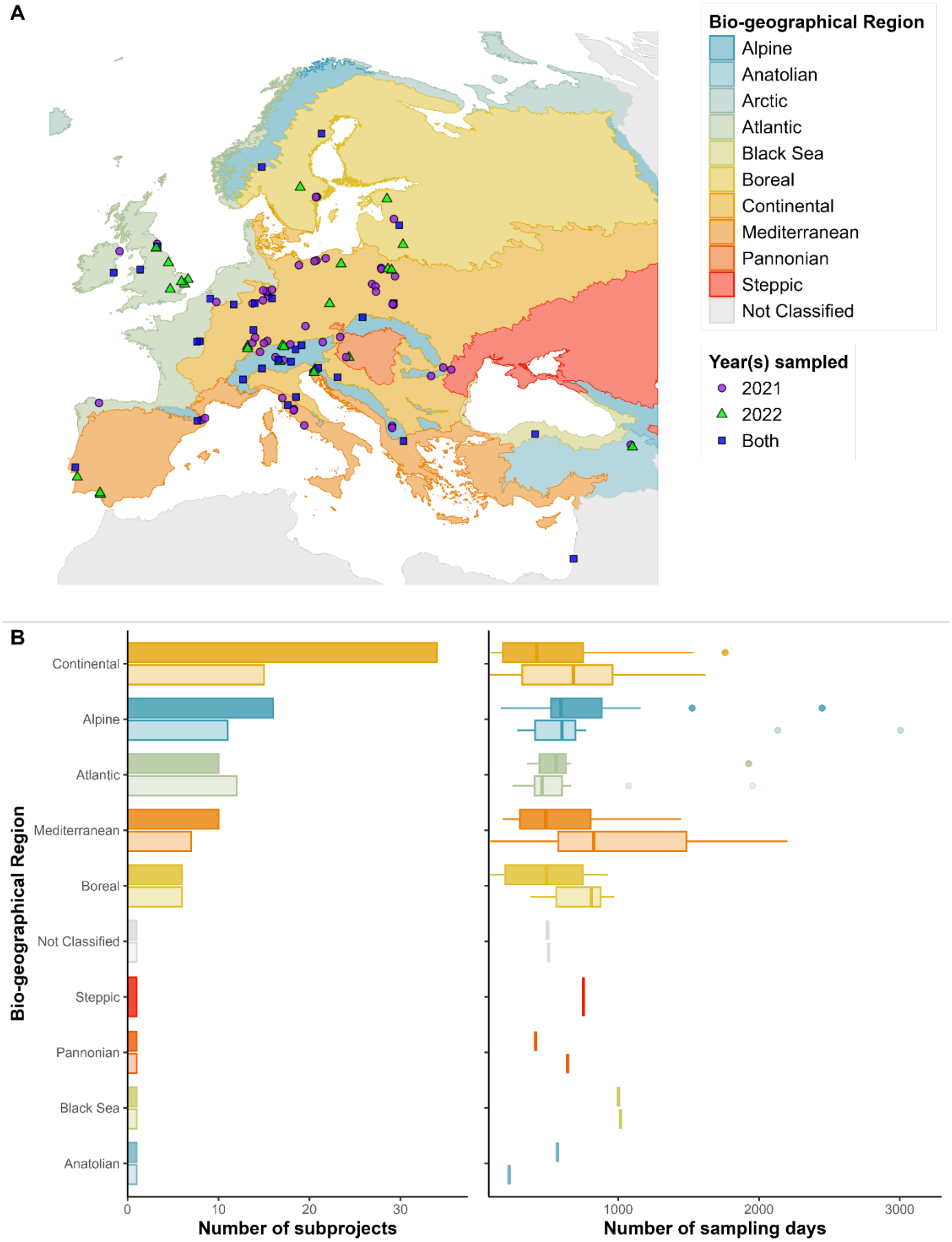

Figure 1. Spatial coverage and sampling effort. A: Map showing the locations of the areas (i.e. subprojects) sampled. The European continent is colour-coded based on the Bio-geographical regions defined by the European Environmental Agency (2016), with colours matching those reported in the y-axis of the plots in B. Shape and colour of the icons indicate whether a subproject was sampled in 2021, 2022, or both years. B: Number of subprojects (left panel) and boxplots of number of sampling days per subproject (right panel) split based

on Bio-geographical regions and years. For each region, bolder and lighter fillings correspond to information related to 2021 and 2022, respectively.

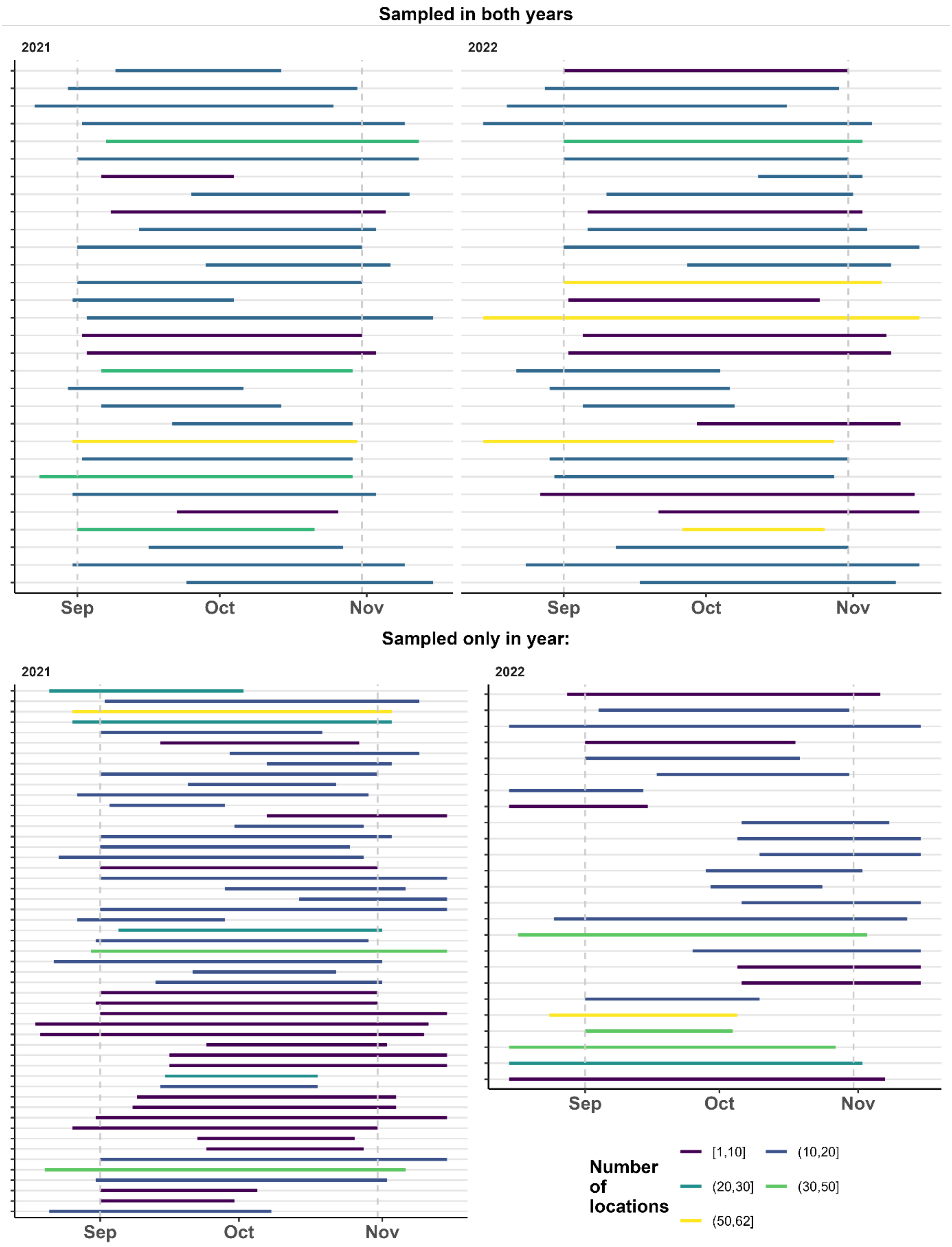

Figure 2. Temporal coverage: sampling periods for each of the subprojects sampled. Top panel: temporal coverage of subprojects sampled in both 2021 and 2022 (i.e. each row

corresponds to a certain subproject). Bottom panels: temporal coverage of subprojects active only in either 2021 (left panel) or 2022 (right panel). Bars are colour-coded based on the number of locations sampled during each year by a specific subproject; vertical, grey, and dashed lines delimit the main period of data collection for the Snapshot Europe initiative, which ranges between September 1st and October 31st of each year. The dataset also includes records collected 2 weeks before (from August 15th) and after (to November 15th) these dates.

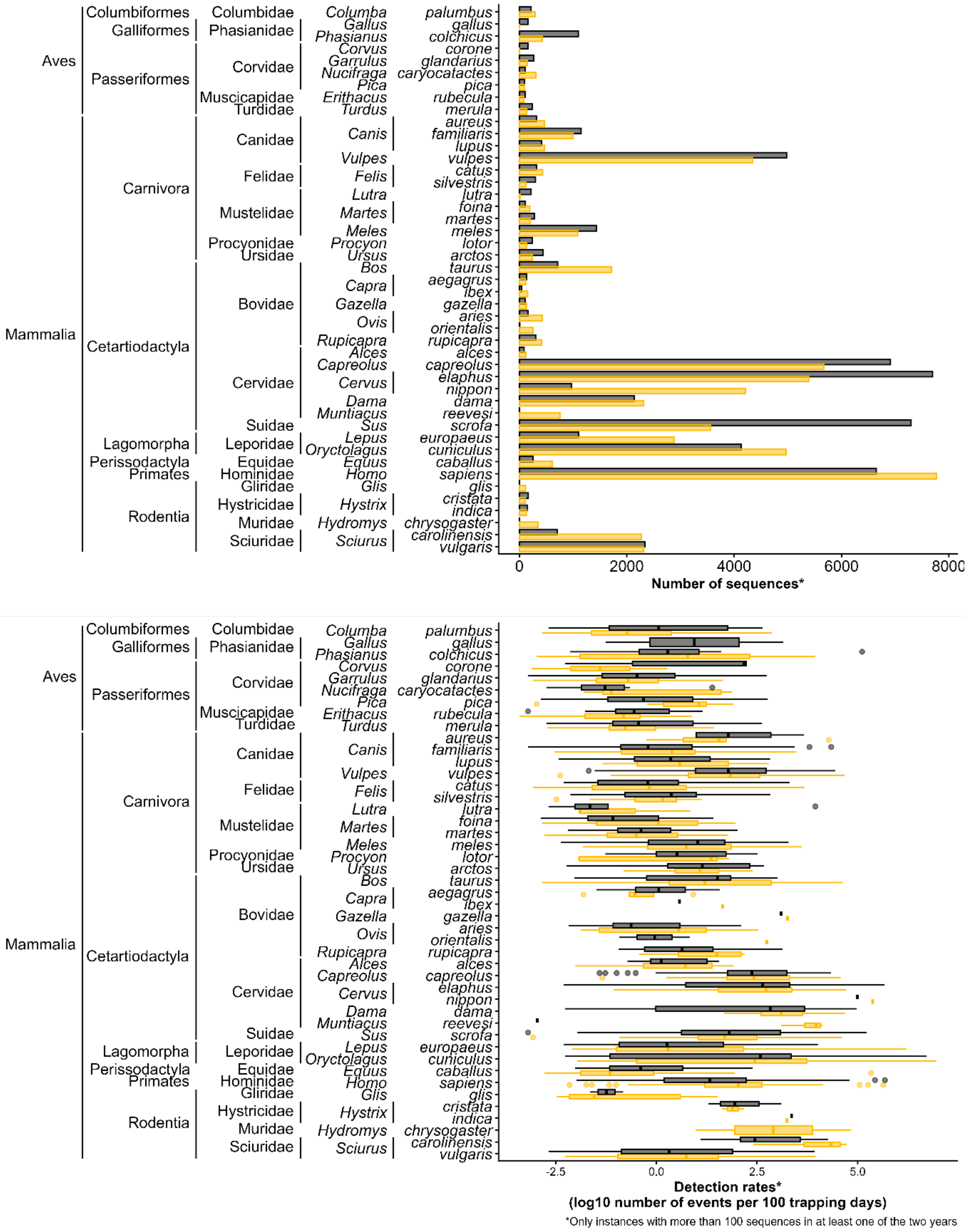

Figure 3. Taxonomic coverage. Total number of sequences (top panel) and detection rates (bottom panel) for all the species of birds and mammals which were detected at least 100 times (i.e. which had at least 100 sequences) in either 2021 (black) or 2022 (yellow). Detection rates were defined per subproject and as the number of events (i.e. sequences, defined as all images taken at a certain location less than 1 minute apart) per 100 camera-trap days. To improve visualisation, the x-axis in the bottom plot is presented in the log10 scale; also *Gallus gallus domesticus* and *Capra aegagrus hircus* are reported in the list of species on the y-axis as *Gallus gallus* and *Capra aegagrus*, respectively.

Table 1. Snapshot Europe 2021–2022's sampling effort quantified as the number of camera-trap days and the number of locations sampled in 2021 and 2022 for each subproject.

**Subproject**	**Number of camera-trap days**		**Number of locations sampled**	
	**2021**	**2022**	**2021**	**2022**
AT_Forest_National Park Hohe Tauern	420	600	12	10
AT_Forest_Wilderness Area Dürrenstein	900	0	22	0
AT_Wetland_Danube National Park	854	0	19	0
BE_Forest_Heidemeersen	421	416	14	14
BE_Wetland_Leuven	1927	0	62	0
CH_Forest_Ettingen BL	0	451	0	10
CH_Forest_Moorlands Ibergeregg	1526	0	23	0
CH_Forest_Parc Naziunal Svizzer	576	0	12	0
CH_Forest_Probstenberg	0	613	0	12
CZ_Forest_PLA Broumovsko	0	682	0	11
DE_Agricultural_Bettenreute	400	0	10	0
DE_Agricultural_District of Goettingen	413	0	13	0
DE_Agricultural_Eschwege	203	0	12	0
DE_Forest_Bavarian National Park	1140	0	19	0
DE_Forest_Berchtesgaden National Park	559	404	19	18
DE_Forest_Black Forest	485	0	16	0
DE_Forest_Black Forest National Park	1072	1088	16	15
DE_Forest_Eifel National Park	1760	1620	29	30
DE_Forest_Goettingen	846	0	14	0
DE_Forest_Hainich National Park	1124	1049	19	19
DE_Forest_Hörre	350	0	14	0
DE_Forest_Jakelberg	0	282	0	6
DE_Forest_Krofdorf	252	264	10	12
DE_Forest_National Park Harz	355	0	10	0
DE_Forest_Oberaula	95	0	14	0
DE_Forest_Schwarzwald	642	0	11	0
DE_Forest_Solling	810	0	15	0
DE_Forest_Stadtwald Konstanz	710	0	11	0
DE_Forest_Trail Langenau	600	0	10	0
DE_Forest_Wetterstein	0	620	0	13
DE_Wetland_Gut Klepelshagen	569	0	17	0
EE_Forest_Turi	0	495	0	12
ES_Agricultural_Pla de Martis	407	0	11	0
ES_Forest_Cantabrian Mountains	352	0	11	0
ES_Forest_Collsacabra	522	672	17	13
ES_ShrubbyVeg_Parque Nacional de Doñana	0	480	0	16
ES_ShrubbyVeg_Parque Nacional de Doñana B	0	93	0	3
ES_Wetland_Aiguamolls Empordà	993	0	16	0
FR_Forest_Nogent	580	580	10	10

FR_Forest_Optmix	500	576	19	20
GB_Agricultural_Carlton Marshes	0	265	0	13
GB_Agricultural_Foxburrow Farm	0	440	0	12
GB_Agricultural_Holywood	354	0	12	0
GB_Agricultural_Lodge Farm	0	414	0	13
GB_Agricultural_RVC Farm	0	380	0	15
GB_Agricultural_Rural	625	0	23	0
GB_Agricultural_Woodland Elwy Valley	631	1075	15	15
GB_Agricultural_Yorkshire	0	250	0	11
GB_Forest_Gosforth Reserve	540	666	16	18
GB_Forest_Pontburn Woods	0	480	0	12
GR_Forest_Nimfeo	900	1712	15	45
HR_Forest_Prolom	582	373	19	8
HU_ShrubbyVeg_Nagy-berek	0	641	0	11
HU_Wetland_Kis-Balaton	413	0	11	0
IE_Forest_County Wicklow	664	1955	19	40
IL_ShrubbyVeg_Ramat Hanadiv Nature Park	499	506	10	10
IT_ArtNonAg_Urban Ecology Varese	299	382	10	10
IT_Forest_Alta Val di Non	1160	0	34	0
IT_Forest_Carline Cornate Fosini	507	827	28	20
IT_Forest_Carso Triestino	442	0	12	0
IT_Forest_Castelporziano	465	0	15	0
IT_Forest_Parco Nazionale Casentino	683	706	20	20
IT_Forest_Parco Regionale Paneveggio	670	609	20	20
IT_Forest_Pejo	0	2134	0	35
IT_Forest_Salbertrand	389	415	11	10
IT_Forest_Stelvio National Park	2449	3005	49	50
IT_OpenAreas_Monte Pisano	268	0	11	0
IT_ShrubbyVeg_Maxcine Tuscany North	180	0	3	0
IT_ShrubbyVeg_Maxcine Tuscany South	242	0	4	0
LT_Forest_Labanoras	0	379	0	13
LV_Forest_Forest Research Station Kalsnava	541	756	19	15
LV_Forest_Ramuli	441	0	8	0
MK_Forest_Mavrovo National Park North	560	0	7	0
MK_Forest_Mavrovo National Park South	165	0	2	0
NO_Forest_Evenstad	926	863	30	15
PL_Forest_Babia Gora National Park	645	470	20	10
PL_Forest_Bialowieza clearing	117	0	3	0
PL_Forest_Biebrza National Park A	170	0	3	0
PL_Forest_Biebrza National Park B	178	0	3	0
PL_Forest_Celestynów District North	765	0	25	0
PL_Forest_Celestynów District South	431	0	13	0
PL_Forest_Gościno Commercial Forest A	168	0	3	0
PL_Forest_Gościno Commercial Forest B	114	0	2	0
PL_Forest_Knyszyńska A	0	120	0	3
PL_Forest_Knyszyńska B	0	80	0	2
PL_Forest_Kozienice Commercial Forest A	228	0	3	0
PL_Forest_Polanów Commercial Forest A	194	0	3	0
PL_Forest_Roztocze National Park A	95	165	3	3
PL_Forest_Roztocze National Park B	99	0	3	0
PL_Forest_Roztocze National Park C	99	0	3	0
PL_Forest_Tuchola Forest	0	747	0	20
PT_ArtNonAg_Montado Companhia Lezirias	1449	1257	30	46
PT_ArtNonAg_Montado Grandola	0	2204	0	55

RO_Forest_Carpathia	879	0	13	0
RO_Forest_Putna-Vrancea Natural Park	1537	0	32	0
RO_Forest_Rezervatia Naturala Padurea Merisor-Cotul Zatuanului	753	0	14	0
SE_Forest_Grimsö	0	880	0	32
SE_Forest_Södermanland East	116	0	4	0
SE_Forest_Södermanland West	87	0	3	0
SE_Forest_Svartberget	820	972	20	20
SI_Forest_Karst	0	871	0	36
SI_Forest_Menisija	588	774	13	12
SI_Forest_Primorska	0	1197	0	30
TR_Forest_Kastamonu	1002	1016	20	20
TR_Forest_Sarıkamış North	569	0	12	0
TR_Forest_Sarıkamış South	0	226	0	3
**Total**	**49061**	**41197**	**1198**	**967**

Table 2. Number of sequences by species and year. Species are grouped as either wild or domestic. Number of sequences depicting humans are reported in the last row.

Class	Family	Genus	Species	Common name	Number of sequences 2021	2022
Wild species						
Aves	Accipitridae			Accipitridae Family	0	1
Aves	Accipitridae	Accipiter		Accipiter Species	1	0
Aves	Accipitridae	Accipiter	gentilis	Northern Goshawk	0	5
Aves	Accipitridae	Accipiter	nisus	Eurasian Sparrowhawk	3	0
Aves	Accipitridae	Buteo		Buteo Species	1	0
Aves	Accipitridae	Buteo	buteo	Eurasian Buzzard	70	17
Aves	Anatidae			Anatidae Family	3	0
Aves	Anatidae	Anas		Anas Species	39	0
Aves	Anatidae	Anser	albifrons	Greater White-fronted Goose	2	0
Aves	Anatidae	Anser	brachyrhynchus	Pink-footed Goose	26	0
Aves	Anatidae	Branta	canadensis	Canada Goose	19	0
Aves	Anatidae	Cygnus	olor	Mute Swan	0	4
Aves	Ardeidae	Ardea	alba	Great White Egret	19	0
Aves	Ardeidae	Ardea	cinereal	Grey Heron	23	2
Aves	Ardeidae	Bubulcus	ibis	Cattle Egret	1	1
Aves	Certhiidae			Certhiidae Family	0	1
Aves	Certhiidae	Certhia	brachydactyla	Short-toed Treecreeper	0	2
Aves	Columbidae			Columbidae Family	2	3
Aves	Columbidae	Columba		Columba Species	1	0
Aves	Columbidae	Columba	livia	Rock Dove	4	0
Aves	Columbidae	Columba	oenas	Stock Dove	2	3
Aves	Columbidae	Columba	palumbus	Common Woodpigeon	220	298
Aves	Columbidae	Spilopelia	senegalensis	Laughing Dove	0	2

Aves	Columbidae	Streptopelia		Streptopelia Species	3	0
Aves	Columbidae	Streptopelia	decaocto	Eurasian Collared-dove	0	1
Aves	Corvidae			Corvidae Family	25	5
Aves	Corvidae	Corvus		Corvus Species	8	4
Aves	Corvidae	Corvus	corax	Common Raven	0	2
Aves	Corvidae	Corvus	cornix	Hooded Crow	10	7
Aves	Corvidae	Corvus	corone	Carrion Crow	165	8
Aves	Corvidae	Corvus	frugilegus	Rook	88	0
Aves	Corvidae	Corvus	monedula	Eurasian Jackdaw	19	1
Aves	Corvidae	Garrulus	glandarius	Jay	267	147
Aves	Corvidae	Nucifraga	caryocatactes	Nutcracker	108	307
Aves	Corvidae	Perisoreus	infaustus	Siberian Jay	0	5
Aves	Corvidae	Pica	pica	Common Magpie	100	91
Aves	Corvidae	Pyrrhocorax	graculus	Yellow-billed Chough	2	14
Aves	Falconidae	Falco		Falco Species	1	0
Aves	Falconidae	Falco	peregrinus	Peregrine Falcon	1	0
Aves	Falconidae	Falco	tinnunculus	Common Kestrel	2	0
Aves	Fringillidae			Fringillidae Family	0	1
Aves	Fringillidae	Carduelis	carduelis	Goldfinch	1	0
Aves	Fringillidae	Fringilla	coelebs	Common Chaffinch	6	6
Aves	Fringillidae	Pyrrhula	pyrrhula	Bullfinch	1	2
Aves	Laridae			Laridae Family	4	0
Aves	Laridae	Larus		Larus Species	4	0
Aves	Laridae	Larus	argentatus	European Herring Gull	42	0
Aves	Laridae	Larus	canus	Mew Gull	36	0
Aves	Laridae	Larus	fuscus	Lesser Black-backed Gull	6	0
Aves	Laridae	Larus	marinus	Great Black-backed Gull	1	0
Aves	Laridae	Larus	ridibundus	Black-headed Gull	4	1
Aves	Motacillidae	Motacilla	cinereal	Grey Wagtail	0	2
Aves	Muscicapidae	Erithacus	rubecula	European Robin	106	82
Aves	Muscicapidae	Oenanthe	oenanthe	Northern Wheatear	0	2
Aves	Muscicapidae	Phoenicurus	ochruros	Black Redstart	3	19
Aves	Muscicapidae	Phoenicurus	phoenicurus	Common Redstart	1	1
Aves	Paridae			Paridae Family	5	1
Aves	Paridae	Cyanistes		Cyanistes Species	1	0
Aves	Paridae	Cyanistes	caeruleus	Eurasian Blue Tit	6	8
Aves	Paridae	Lophophanes	cristatus	Crested Tit	1	0
Aves	Paridae	Parus	major	Great Tit	47	27
Aves	Paridae	Parus	minor	Eastern Great Tit	0	5
Aves	Paridae	Periparus	ater	Coal Tit	1	0
Aves	Paridae	Poecile	montanus	Willow Tit	0	1
Aves	Passeridae			Passeridae Family	11	0
Aves	Passeridae	Passer		Passer Species	0	1

Aves	Phasianidae			Phasianidae Family	3	67
Aves	Phasianidae	Alectoris	chukar	Chukar	22	13
Aves	Phasianidae	Alectoris	graeca	Rock Partridge	1	0
Aves	Phasianidae	Alectoris	rufa	Red-legged Partridge	3	9
Aves	Phasianidae	Bonasa	bonasia	Hazel Grouse	13	3
Aves	Phasianidae	Lagopus	lagopus	Willow Grouse	3	0
Aves	Phasianidae	Lyrurus	tetrix	Black Grouse	7	0
Aves	Phasianidae	Perdix	perdix	Grey Partridge	8	1
Aves	Phasianidae	Phasianus		Phasianus Species	1	0
Aves	Phasianidae	Phasianus	colchicus	Ring-necked Pheasant	1098	426
Aves	Phasianidae	Syrmaticus	reevesii	Reeves's Pheasant	24	0
Aves	Phasianidae	Tetrao	urogallus	Western Capercaillie	27	14
Aves	Picidae			Picidae Family	0	1
Aves	Picidae	Dendrocopos		Dendrocopos Species	0	2
Aves	Picidae	Dendrocopos	major	Great Spotted Woodpecker	16	9
Aves	Picidae	Dryocopus	martius	Black Woodpecker	26	10
Aves	Picidae	Picus	viridis	Green Woodpecker	18	2
Aves	Prunellidae	Prunella	collaris	Alpine Accentor	0	2
Aves	Rallidae	Gallinula	chloropus	Moorhen	0	1
Aves	Rallidae	Porzana	porzana	Spotted Crake	1	0
Aves	Scolopacidae	Scolopax	rusticola	Eurasian Woodcock	16	2
Aves	Sittidae	Sitta	europaea	Nuthatch	1	4
Aves	Strigidae			Strigidae Family	5	0
Aves	Strigidae	Bubo	bubo	Eagle Owl	0	1
Aves	Strigidae	Strix	aluco	Tawny Owl	7	19
Aves	Sturnidae	Acridotheres	tristis	Common Myna	1	1
Aves	Sturnidae	Sturnus	vulgaris	Common Starling	4	0
Aves	Troglodytidae	Troglodytes	troglodytes	Wren	4	9
Aves	Turdidae			Turdidae Family	0	28
Aves	Turdidae	Turdus		Turdus Species	103	10
Aves	Turdidae	Turdus	iliacus	Redwing	4	0
Aves	Turdidae	Turdus	merula	Common Blackbird	243	134
Aves	Turdidae	Turdus	philomelos	Song Thrush	72	51
Aves	Turdidae	Turdus	pilaris	Fieldfare	3	0
Aves	Turdidae	Turdus	torquatus	Ring Ouzel	0	4
Aves	Turdidae	Turdus	viscivorus	Mistle Thrush	19	5
Aves	Tytonidae	Tyto	alba	Common Barn Owl	18	1
Mammalia	Bovidae			Bovidae Family	1	0
Mammalia	Bovidae	Bison	bonasus	European Bison	38	1
Mammalia	Bovidae	Capra		Capra Species	0	3
Mammalia	Bovidae	Capra	ibex	Alpine Ibex	43	155
Mammalia	Bovidae	Gazella	gazelle	Mountain Gazelle	110	131
Mammalia	Bovidae	Ovis	orientalis	Mouflon	9	252
Mammalia	Bovidae	Rupicapra	rupicapra	Northern Chamois	303	410

Mammalia	Canidae			Canine Family	34	36
Mammalia	Canidae	Canis		Canis Species	22	8
Mammalia	Canidae	Canis	aureus	Golden Jackal	317	466
Mammalia	Canidae	Canis	lupus	Grey Wolf	417	468
Mammalia	Canidae	Nyctereutes		Nyctereutes Species	1	0
Mammalia	Canidae	Nyctereutes	procyonoides	Raccoon Dog	52	34
Mammalia	Canidae	Vulpes	vulpes	Red Fox	4976	4338
Mammalia	Castoridae	Castor	fiber	Eurasian Beaver	3	3
Mammalia	Cervidae			Cervidae Family	434	422
Mammalia	Cervidae	Alces	alces	Moose/Elk	88	124
Mammalia	Cervidae	Capreolus	capreolus	European Roe Deer	6911	5670
Mammalia	Cervidae	Cervus		Cervus Species	5	19
Mammalia	Cervidae	Cervus	elaphus	Red Deer	7695	5387
Mammalia	Cervidae	Cervus	nippon	Sika Deer*	973	4207
Mammalia	Cervidae	Dama	dama	Common Fallow Deer	2137	2317
Mammalia	Cervidae	Hydropotes	inermis	Water Deer	0	59
Mammalia	Cervidae	Muntiacus	reevesi	Reeves' Muntjac	1	756
Mammalia	Cricetidae			Cricetidae Family	11	0
Mammalia	Cricetidae	Microtus	arvalis	Common Vole	1	0
Mammalia	Cricetidae	Myodes		Myodes Species	1	0
Mammalia	Erinaceidae	Erinaceus	concolor	Southern White-breasted Hedgehog	4	21
Mammalia	Erinaceidae	Erinaceus	europaeus	Western European Hedgehog	28	54
Mammalia	Felidae			Cat Family	4	1
Mammalia	Felidae	Felis		Felis Species	72	31
Mammalia	Felidae	Felis	silvestris	European Wildcat	300	118
Mammalia	Felidae	Lynx	lynx	Eurasian Lynx	66	14
Mammalia	Gliridae	Eliomys	quercinus	Garden Dormouse	13	15
Mammalia	Gliridae	Glis	glis	Edible Dormouse	2	115
Mammalia	Herpestidae	Herpestes	ichneumon	Egyptian Mongoose	17	24
Mammalia	Hyaenidae	Hyaena	hyaena	Striped Hyaena	6	0
Mammalia	Hystricidae	Hystrix	cristata	Crested Porcupine	167	104
Mammalia	Hystricidae	Hystrix	indica	Indian Crested Porcupine	143	130
Mammalia	Leporidae			Rabbit and Hare Family	464	45
Mammalia	Leporidae	Lepus		Lepus Species	156	92
Mammalia	Leporidae	Lepus	corsicanus	Corsican Hare	26	0
Mammalia	Leporidae	Lepus	europaeus	European Hare	1106	2880
Mammalia	Leporidae	Lepus	granatensis	Iberian Hare	16	29
Mammalia	Leporidae	Lepus	timidus	Mountain Hare	30	41
Mammalia	Leporidae	Oryctolagus	cuniculus	European Rabbit	4131	4968
Mammalia	Muridae			Muridae Family	124	6
Mammalia	Muridae	Apodemus		Apodemus Species	190	12
Mammalia	Muridae	Apodemus	agrarius	Striped Field Mouse	4	0
Mammalia	Muridae	Apodemus	sylvaticus	Wood Mouse	95	23

Mammalia	Muridae	Hydromys	chrysogaster	Common Water Rat	0	346
Mammalia	Muridae	Rattus		Rattus Species	1	15
Mammalia	Muridae	Rattus	norvegicus	Brown Rat	45	22
Mammalia	Muridae	Rattus	rattus	House Rat	4	0
Mammalia	Mustelidae			Weasel Family	163	34
Mammalia	Mustelidae	Lutra	lutra	Eurasian Otter	220	17
Mammalia	Mustelidae	Martes		Martes Species	390	303
Mammalia	Mustelidae	Martes	foina	Beech/Stone Marten	113	195
Mammalia	Mustelidae	Martes	martes	European Pine Marten	283	199
Mammalia	Mustelidae	Meles	meles	Eurasian Badger	1435	1085
Mammalia	Mustelidae	Mustela		Weasel Species	10	3
Mammalia	Mustelidae	Mustela	erminea	Stoat	22	9
Mammalia	Mustelidae	Mustela	nivalis	Least Weasel	3	0
Mammalia	Mustelidae	Mustela	putorius	Western Polecat	13	15
Mammalia	Myocastoridae	Myocastor	coypus	Nutria	0	13
Mammalia	Procyonidae	Procyon	lotor	Northern Raccoon	245	132
Mammalia	Rhinolophidae	Rhinolophus		Rhinolophus Species	4	0
Mammalia	Sciuridae			Sciuridae Family	137	33
Mammalia	Sciuridae	Marmota	marmota	Alpine Marmot	13	15
Mammalia	Sciuridae	Sciurus		Sciurus Species	17	9
Mammalia	Sciuridae	Sciurus	anomalus	Caucasian Squirrel	2	0
Mammalia	Sciuridae	Sciurus	carolinensis	Eastern Gray Squirrel	705	2268
Mammalia	Sciuridae	Sciurus	vulgaris	Eurasian Red Squirrel	2342	2308
Mammalia	Suidae			Suidae Family	0	1
Mammalia	Suidae	Sus	scrofa	Wild Boar	7296	3556
Mammalia	Ursidae	Ursus	arctos	Brown Bear	445	244
Mammalia	Vespertilionidae	Nyctalus		Nyctalus Species	2	0
Mammalia	Vespertilionidae	Plecotus		Plecotus Species	1	0
Mammalia	Viverridae	Genetta	genetta	Common Genet	55	56
Domestic species						
Aves	Phasianidae	Gallus	gallus domesticus	Domestic Chicken	158	0
Mammalia	Bovidae	Bos	taurus	Domestic Cattle	713	1714
Mammalia	Bovidae	Bubalus	bubalis	Domestic Water Buffalo	5	0
Mammalia	Bovidae	Capra	aegagrus hircus	Domestic Goat	137	116
Mammalia	Bovidae	Ovis	aries	Domestic Sheep	166	424
Mammalia	Canidae	Canis	familiaris	Domestic Dog	1150	997
Mammalia	Equidae	Equus	asinus	Domestic Donkey	90	20
Mammalia	Equidae	Equus	caballus	Domestic Horse	253	611
Mammalia	Felidae	Felis	catus	Domestic Cat	318	434
Mammalia	Suidae	Sus	scrofa scrofa	Domestic Pig	0	30
Humans						
Mammalia	Hominidae	Homo	sapiens	Human	6647**	7767**

*This include an unknown proportion of *red deer x sika deer* hybrids. Phenotypically-sika deer were classified as sika, and phenotypic hybrids were generally tagged as *Cervus sp*.
**These values do not include sequences taken at cameras' deployment and retrieval; however, these records are included in the dataset (e.g., to assess the impact of human disturbance).

6. Sources of funding: Data were collected thanks to the volunteer work of researchers across Europe. Coordination and management were provided by the Max Planck Institute of Animal Behavior. FI received funding from the European Union's Horizon 2020 research and innovation programme under the Marie Skłodowska-Curie grant agreement No 101107666. For site-specific arrays, additional support was provided by the following:

BE_Wetland_Leuven: This work makes use of data and/or infrastructure provided by INBO and funded by Research Foundation - Flanders (FWO) as part of the Belgian contribution to LifeWatch; DE_Agricultural_District of Goettingen: Lower Saxony Ministry of Food, Agriculture and Consumer Protection; DE_Forest_Schwarzwald: NM received funding from the EU's Horizon 2020 research and innovation programme under the Marie Sklodowska-Curie grant agreement No 894290 - Part of this study was funded by the German Research Foundation within the Research Training Group ConFoBi (grant number GRK 2123/2); EE_Forest_Turi: Funding: LIFE18IPE/EE/000007; EE_Forest_Turi: Icelandic Centre for Research (RANNIS; grant 217753); FR_Forest_Nogent and FR_Forset_Optmix: The experimental site OPTMix (https://optmix.inrae.fr) was installed and equipped by INRAE EFNO thanks to the Centre Val-de-Loire region, the Loiret and the French National Forest Office, and belongs to networks ANAEE-F (http://www.anaee-france.fr/); GB_Agricultural_Yorkshire: University of Hull; HU_Wetland_Kis-Balaton and HU_ShrubbyVeg_Nagy-berek: The work of JL was supported by the OTKA K 145933 grant (National Research, Development and Innovation Office – NKFIH); IT_Forest_Parco Nazionale Casentino: IG was supported by the European Union-Next Generation EU's National Biodiversity Future Centre, funded by the Italian Ministry of University and Research, PNRR, while field activities in Parco Nazionale Foreste Casentinesi have been funded by the park itself; PL_Forest_Knyszynska A and other subprojects from Poland: The work of ZB was supported by funding from the Polish Ministry of Science (grant number 900-418); PL_Forest_Tuchola Forest: the work of DPJK, KK, ES-S was supported by funding of the National Science Centre, Poland (grant number 2021/41/B/NZ8/00015); PL_Forest_Babia Gora National Park: the work by IW was partially funded by the research grant from the Jagiellonian University, grant number N18/DBS/000003; RO_Forest_Putna-Vrancea Natural Park: Romanian National Authority for Scientific Research (grant PN-III-P1-1.1-TE-2019- 0835); SE_Forest_Södermanland East, SE_Forest_Södermanland West: Swedish Research Council for Sustainable Development (FR-2020/0008) and the Swedish Environmental Protection Agency (GD-2022-0003); SI_Forest_Menisija: MK was supported by the Slovenian Research and Innovation Agency (grant J1-50013) and Biodiversa+ (BIG_PICTURE project). Study was co-funded by the European Commission under the LIFE Programme (LIFE16 NAT/SI/000634 LIFE Lynx project); SI_Forest_Primorska: The Slovenian Research and Innovation Agency (ARIS; programme groups P1-0386; project N1-0281) and the STEPCHANGE European Union's Horizon 2020 Research and Innovation Program under grant agreement No. 101006386; TR_Forest_Kastamonu: Republic of Turkiye Ministry of Agriculture and Forestry General Directorate of Nature Conservation and National Parks.

B. Specific subproject description

1. Site description

a. Site type: The data presented here were collected at 81 and 55 arrays (i.e. subprojects, clusters of camera-trap sites) spread across 22 and 23 countries in 2021 and 2022, respectively. Thirty (28%) of these unique 106 arrays were sampled in both years (Fig. 1A).

b. Geography: The locations sampled ranged from south to north (latitudinal range, 2021: 32.53664 to 64.24829; 2022: 32.53642 to 64.25326) and east to west (longitudinal range, 2021: -8.83323 to 42.52857; 2022: -8.80143 to 42.62157) Europe (Fig. 1A). An array from Israel is also included in the dataset.

c. Habitat: The areas sampled encompass a large variety of habitat types, from alpine regions in the north to Mediterranean biomes in the south. Out of the 12 biogeographical regions identified by the European Environmental Agency (2016), which defines the operative units for the Habitat Directive, the dataset contains data from 10 regions; only Arctic and Macaronesia are missing (Fig. 1B). The areas sampled encompassed all the major natural and semi-natural terrestrial environments that occur in Europe (Fig. 1B). The list includes natural environments ranging from forests to open wetlands, pastures, and shrubby vegetation, and human-modified environments from agricultural to green urban areas.

d. Geology, landform: Altitude varied greatly (2021: [2 - 2,701]; 2022: [0 - 2,432] m a.s.l.), ranging from locations almost at sea level in Belgium, Spain and Portugal to high elevation in the Italian and Swiss Alps and Turkey.

2. Experimental or sampling design

a. Design characteristics: Collaborators were asked to collect data for a minimum of 400 camera-trap days per array during September and October of each year at a number of sites between 10 and 50. However, due to camera failures and other logistic constraints, the final number of locations per array ranged from 2 to 62, and the sampling effort ranged from 80 to 3,005 camera-trap days (Table 1). Each team (composed of 1 to 8 people, typically 2 to 3) collected data at one or multiple arrays (typically 1) located in areas of their choice and was asked to describe each of their arrays by choosing a combination of habitat (agricultural areas, artificial non-agricultural vegetated areas i.e. gardens and parks, shrubby vegetation, pastures and natural grassland, forest, open areas i.e. beaches, burnt areas and glaciers, wetland) and settings (Urban, Suburban, Rural, Wild, Other) descriptors. Each array was named based on these descriptors following the format 2-letter code country, habitat type, array-specific name, and year (e.g. 'DE_Forest_Stadtwald Konstanz_21'). All sites within an array were then chosen by the team to match the selected habitat-settings combination. In an array, each site had to be more than 200 m apart from all the others, but less than 5 km away from at least another camera-trap site. No requirement was defined in relation to the shape of the array (e.g. regular grid, transect) or the selection of sites (e.g. randomized) within an array. Camera-trap models were restricted to those with infrared (IR) flash, a trigger speed less or equal to 0.5 seconds, and models that could fire consecutive bursts of pictures without

a delay between subsequent triggers. These decisions were made intentionally to allow as many researchers as possible to contribute and participate, while still ensuring the robustness of the data collected. We invite future users of the data to carefully evaluate these characteristics of the dataset in relation to the goals of their analyses and the statistical methodologies adopted. For example, the range of distance among the sites sampled within an array (between 200 m and 5 km) might provide a challenge in some statistical frameworks (e.g. using the classic occupancy modeling approach: MacKenzie et al., 2002) while it can be readily accommodated in others (e.g. using a spatially explicit occupancy modeling approach: Doser et al., 2022). Similarly, although applying a 1-minute threshold to define sequences (i.e. independent events) reduces temporal autocorrelation in the observations, some analyses may require additional temporal aggregation (e.g., using the maximum number of individuals within 30-minute intervals) to further minimise temporal dependence in the data. The dataset also includes records collected in 22 subprojects that did not meet the minimal requirements in terms of number of locations and camera trap days sampled; the choice of including these records was made to maximise the amount of information shared. Again, future users might subset the dataset based on the specific goals and requirements of their own analyses. Additionally, we stress that the protocol used was not developed to specifically target bird species that are rarely active on or in proximity to the ground; thus, although still useful, the records of birds present in the dataset might not be representative of the full bird diversity present in a certain area, but only of a portion of species that can readily be detected based on the protocol adopted.

The sampling protocol was originally developed by Roland Kays, William McShea, and Michael Cove and follows the requirements already applied for previous years of the Snapshot USA (Cove et al. 2021, Kays et al. 2022, Shamon et al. 2024, Rooney et al. 2025) and Snapshot Japan (Fukasawa et al. 2025) initiatives.

b. Permanent plots: Thirty (28%) of the 106 arrays uniquely represented in this dataset were sampled in both years (Figs. 1A and 2).

c. Data collection period, frequency, etc.: Around 91% of the data were collected from September 1st to October 31st of each year (Fig. 2). Data collected two weeks before and after this period were also included in the dataset. See Class II section A3 for the exact extent of the period of sampling in each year.

3. Research methods

a. Field/laboratory: We asked collaborators to take a picture when setting and retrieving the cameras and annotate location coordinates (in longitude and latitude), start and end date of sampling (i.e. date of retrieval or date of camera-trap failure, if applicable), camera make and model for each deployment (i.e. a camera set at a location for a certain period). The use of baits or lures was not allowed. When cameras were placed along trails or logging roads, this information was recorded and included in the dataset. With few exceptions to adapt to particular setting conditions (e.g. camera site on slopes), all cameras were placed ~50 cm above the ground (knee height), parallel to the ground with horizontal orientation, pointing North to avoid false triggers due to sun glare, and attached to sturdy trees or other structures. Cameras were set to take a burst of at least 3 images per trigger.

b. Instrumentation: Each team was responsible for uploading and processing their data in Wildlife Insights (https://www.wildlifeinsights.org/). Members of each team revised and edited the image classification provided by the AI model within the Wildlife Insights

platform (https://github.com/google/cameratrapai; Gadot et al. 2024). The leader of each team was ultimately responsible for revising the classifications made by members of their team. The Snapshot Europe core team reviewed a random sample of these classifications: around 10% (~13,000) of the non-empty sequences and around 2% (~2,500) of the empty sequences. The core team also reviewed sequences with 'suspicious' tags (e.g. species not expected to be found in the areas sampled) and sequences assigned to coarser taxonomic groups, when only one possible species was present in the area (e.g. 'Ursidae' were reclassified as '*Ursus arctos*'). During upload, images were automatically organized in sequences, such that each sequence contained all the images collected at a certain location less than 1 minute apart from the previous and following images. The classification was done at the sequence level (e.g. recording all the species identifiable in a sequence) and at the lowest taxonomic level possible without doubts. Non-target species (e.g. reptiles, insects) were usually recorded at the class level; these observations were not included in this dataset because of being too sparse. Collaborators were not required to record information beyond the species present in a sequence, for lack of standardization in classifying some information (e.g. age) or difficulties in determining others (e.g. gender) in images. However, some collaborators reported this information based on their expertise. All the pictures in sequences containing one or more images of humans were automatically replaced by a placeholder (i.e. a black image), whereas the associated information on the presence of people at the related place and time was stored in the dataset under generic labels (species: "*Homo sapiens*"; tags; "Human-Camera-trappers", "Human-bikers", "Human-pedestrian", etc).

We downloaded the data from Wildlife Insights (wildlifeinsights.org) as .csv files on 26 February 2026 (projects 'Snapshot Euro 2021': Iannarilli et al. 2025a; and 'Snapshot Euro 2022': Iannarilli et al. 2025b). We first corrected a few remaining issues within each individual dataset and then merged the two to obtain the compiled version of the dataset associated with this publication. These final steps were performed in R (R Core Team 2022). The code related to cleaning the data and recreating the figures, tables, and summary statistics provided in this document is publicly available at https://doi.org/10.5281/zenodo.21066894.

c. Taxonomy and systematics: We adopted the taxonomic convention used by Wildlife Insights (https://www.wildlifeinsights.org/get-started/taxonomy#taxonomy). As such, we used the taxonomies defined by the IUCN Red List of Endangered Species (https://www.iucnredlist.org/) and the American Society of Mammalogists Mammal Diversity Database (https://www.mammaldiversity.org/) for mammals, and the taxonomy defined by Birdlife International (https://datazone.birdlife.org/species/taxonomy) for birds.

d. Permit history: NA

e. Legal/organizational requirements: In compliance with the European General Data Protection Regulation (GDPR) regulating the collection and ethical use of personal information, no information (e.g. gender, age) that might lead to the identification of the people in the images was recorded. All images of sequences containing pictures of people were automatically replaced by a placeholder (i.e. a black image) and only generic labels (species: "*Homo sapiens*"; tags; "Human-Camera-trappers", "Human-bikers", "Human-pedestrian", etc) were retained in the dataset to record the presence of humans at a certain location and time. Recording information on the presence and frequency of site use by humans enables studies on the effect of anthropogenic disturbance at fine spatio-temporal scales (Burton et al. 2024).

4. Project personnel: The core team (listed in Class I section C1) launched the call for participation in the Snapshot Europe initiative, provided resources to coordinate its activities and ensure data quality and dataset coherence. All authors and their teams collected and contributed data and/or assisted with species classification of the images, expert review, and data cleaning. The initiative was supported by the Max Planck Institute of Animal Behavior in partnership with EuroMammals and Snapshot USA.

Class III. Data set status and accessibility

A. Status

1. Latest update: 2 March 2026

2. Latest archive date: 2 March 2026

3. Metadata status: 2 March 2026, version submitted

4. Data verification: Members of the core team revised the data through summary graphics and routine checks on issues commonly occurring in camera-trap datasets (e.g. typos in location coordinates and start and end dates of deployments). When an issue was identified, the core team reached out to the lead team responsible for those specific data for clarification. We further required collaborators to review their data in the final version of the dataset.

B. Accessibility

1. Storage location and medium: In addition to this publication, this dataset is also available at Edmond – the Open Research Data Repository of the Max Planck Society: https://doi.org/10.17617/3.7YLWMU. The related images are accessible through Wildlife Insights (wildlifeinsights.org; projects ‘Snapshot Euro 2021’: Iannarilli et al. 2025a; and ‘Snapshot Euro 2022’: Iannarilli et al. 2025b) by submitting a data download request or filtering for the related project in the ‘Explore data’ tab. Information on the spatial, temporal and taxonomic coverage of the data can be previewed at https://fabiolaiannarilli.shinyapps.io/snapshot_europe_2021_2022_data_exploration_shiny/.

2. Contact persons: Fabiola Iannarilli (email: fiannarilli@ab.mpg.de), Max Planck Institute of Animal Behavior, Am Obstberg 1, 78315 Radolfzell am Bodensee, Germany.

3. Copyright restrictions: The data are available for reuse under CC BY-SA 4.0 licensing.

4. Proprietary restrictions: When using this dataset for publication, please cite this paper and, if applicable, the relative projects within Wildlife Insights:

- Iannarilli, Fabiola; Keeves-von Wolff, Brigitta; Wikelski, Martin; Heurich, Marco; Kays, Roland; Cagnacci, Francesca. 2021. Last updated February 2025. Snapshot Europe 2021. http://n2t.net/ark:/63614/w12003388. Accessed via wildlifeinsights.org on 2026-02-26.
- Iannarilli, Fabiola; Keeves-von Wolff, Brigitta; Wikelski, Martin; Heurich, Marco; Kays, Roland; Cagnacci, Francesca. 2022. Last updated February 2025. Snapshot Europe 2022. http://n2t.net/ark:/63614/w12004446. Accessed via wildlifeinsights.org on 2026-02-26.

a. Release date: 2 March 2026

b. Citation: Please, see section III.B.4 ‘Proprietary restrictions’.

c. Disclaimer(s): None

5. Costs: None.

Class IV. Data structural descriptors

A. Data set file

1. Identity: DataS1.zip contains:

   Snapshot_Europe_2021_2022_cameras.csv;
   Snapshot_Europe_2021_2022_deployments.csv;
   Snapshot_Europe_2021_2022_projects.csv;
   Snapshot_Europe_2021_2022_sequences.csv;
   Snapshot_Europe_2021_2022_images.csv

2. Size: Total size: 134.72 MB (zip)

   Snapshot_Europe_2021_2022_cameras.csv: 1198 rows, 8 columns, 48 KB;
   Snapshot_Europe_2021_2022_deployments.csv: 2596 rows, 31 columns, 795 KB;
   Snapshot_Europe_2021_2022_projects.csv: 3 rows, 28 columns, 2 KB;
   Snapshot_Europe_2021_2022_sequences.csv: 126460 rows, 27 columns, 3.1 MB;
   Snapshot_Europe_2021_2022_images.csv: 1466069 rows, 16 columns, 59.0 MB.

3. Format and storage mode: comma-separated values (.csv)

4. Header information: See column descriptions in section B

5. Alphanumeric attributes: Mixed

6. Special characters/fields: None

7. Authentication procedures: None

B. Variable information

The data format follows that used by Wildlife Insights for data download. As such, here we expand on their Data Dictionary (provided with each data download), adding information on additional variables included in the dataset (e.g. ‘year’) and further details on the data type.

Table 3. Variable information for the file Snapshot_Europe_2021_2022_cameras.csv, which contains details on the camera devices used to collect the data.

**Variable identity**	**Variable definition**	**Data type**
year	Identifier for specific Snapshot Europe survey, either 2021 or 2022	Integer. Potential values: 2021 and 2022

project_id	An internal identification for the project.	Integer. Potential values: 2003388, 2004446
camera_id	An internal identification for a camera trap	Character
camera_name	An internal identification for a camera trap	Character
make	Manufacturer of the camera	Character. Missing value codes: ""
model	The model of the camera	Character. Missing value codes: ""
serial_number	The serial number of the camera trap.	Character. Missing value codes: NA
year_purchased	The date the camera was purchased.	Character. Missing value codes: NA

Table 4. Variable information for the file Snapshot_Europe_2021_2022_deployments.csv, which contains details on deployment location, duration, and potential attractants or site features that might have enhanced species encounter during sampling.

**Variable identity**	**Variable definition**	**Data type**
year	Identifier for specific Snapshot Europe survey, either 2021 or 2022	Integer. Potential values: 2021, 2022
project_id	An internal identification for the project.	Integer. Potential values: 2003388, 2004446
deployment_id	A unique identifier for the camera deployment (within the project).	Character
placename	A name for the location.	Character
longitude	Longitude of Location in decimal degrees using datum WGS84.	Integer. Number of significant digits: 6
latitude	Latitude of Location in decimal degrees using datum WGS84.	Integer. Number of significant digits: 5
start_date	The date the camera was activated and starting recorded observations. This also marks the date a new SD card was inserted into a camera that is continuously operating.	Date. Format: YYYY-MM-DD
end_date	The date the camera was picked up OR the date the SD card was retrieved. Alternatively, the date the camera stopped functioning. Whatever comes	Date. Format: YYYY-MM-DD

	earlier.	
bait_type	Type of bait (if any) that was used with the camera deployment.	Character. Potential values: None, Scent, Meat, Visual, Acoustic, Other
bait_description	Text field to describe baiting method and Other bait type if necessary	Character
feature_type	Type of feature (if any) that the camera deployment is associated with.	Character. Potential values: None, Road paved, Road dirt, Trail hiking, Trail game, Road underpass, Road overpass, Road bridge, Culvert, Burrow, Nest site, Carcass, Water source, Fruiting tree, Other
feature_type_methodology	Text field to describe details about a feature type.	Character. Missing value codes: NA
camera_id	An internal identification for a camera trap.	Integer
camera_name	A user-defined name for a camera trap.	Character
quiet_period	Time specified between shutter triggers when activity in the sensor will not trigger the shutter.	Integer. Units of measurement: seconds
camera_functioning	Details the status of the camera after the named deployment.	Character. Potential values: Camera Functioning, Unknown Failure, Vandalism, Theft, Memory Card, Film Failure, Camera Hardware Failure, Wildlife Damage
sensor_height	The height at which the sensor was deployed.	Character. Potential values: Chest height, Knee height, Canopy, Unknown, Other
height_other	Text field to describe sensor height if "other" is selected.	Character. Free text only when 'other' selected for Height or Angle
sensor_orientation	Text field to describe sensor angle if "other" is selected.	Character. Potential values: Parallel, Pointed Downward, Varies, Unknown, Others
orientation_other	Text field to describe sensor angle if "other" is selected.	Character. Free text only when 'other' selected for Height or Angle. Missing value codes: NA
plot_treatment	A parcel of land defined by a specific function, property, or purpose. Examples include types of agriculture anddifferent stages of controlled burns.	Character. Missing value codes: NA

plot_treatment_description	General description of the plot treatment.	Character. Missing value codes: NA
detection_distance	Maximum distance at which a camera triggered, as tested during deployment, measured in meters.	Integer. Missing value codes: NA
subproject_name	A name for a logical grouping of deployments within a project. Subprojects are similar to Events and Arrays, but do not necessarily need to be grouped by time or theme.	Character
subproject_design	Text field to describe subproject design methods.	Character. Missing value codes: NA
event_name	Camera-trap arrays can be grouped into sampling `events` (not to be confused with events of animal detection by camera traps). There are many uses for an event and it is designed to be flexibly used. Common events could be seasons (wet and dry), months, years or other types of logical groupings when field sampling occurs.	Character. Missing value codes: NA
event_description	A description that defines the events.	Character. Missing value codes: NA
event_type	A broader category for types of events. For example, some projects may put sensors out using different sampling designs in the wet season vs the dry season. This is a 'Seasonal' event type and will make grouping data together for data management.	Character. Missing value codes: NA
recorded_by	The person installing the camera.	Character. Missing value codes: NA
remarks	Remarks about the deployment	Character. Missing value codes: NA
subproject_name_no_year	subproject_name excluding the last three characters (i.e. either '_21' or '_22') which specify the year of data collection	Character

Table 5. Variable information for the Snapshot_Europe_2021_2022_projects.csv file, which reports details to characterize the project (e.g. project name, objective, target species).

**Variable identity**	**Variable definition**	**Data type**
year	Identifier for specific Snapshot Europe survey, either 2021 or 2022	Integer. Potential values: 2021, 2022

project_id	An internal identification for the project.	Integer. Potential values: 2003388, 2004446
project_name	A name that identifies the project.	Character
project_short_name	A short name that identifies the project.	Character
project_objectives	The goals of the project.	Character
project_species	If the study's objective is to focus on single or multiple species.	Character. Potential values: Individual, Multiple
project_species_individual	If the study focuses on an individual species, which species?	Logic. Missing value codes: NA
project_sensor_layout	The sensor layout or sampling design within the study area.	Character. Potential values: Systematic, Randomized, Convenience, Targeted, Unknown
project_sensor_layout_targeted_type	If the sensor layout is targeted, what wildlife sign or other feature was used?	Character.
project_bait_use	If sensors were baited at all or some of the deployment locations.	Character. Potential values: Yes, Some, No
project_bait_type	If sensors were baited, what bait was used?	Character. Potential values: None, Scent, Meat, Visual, Acoustic, Other
project_stratification	If sensors are distributed along different habitat types, legal zones (e.g. protected area vs. not protected) or other biophysical features (e.g. elevation, rainfall, etc.)?	Character. Potential values: Yes, No
project_stratification_type	If sensors are stratified, the strata is listed here.	Character
project_sensor_method	If sampling is based on motion sensor detection, timelapse, or both.	Character. Potential values: Sensor Detection, Time Lapse, Both
project_individual_animals	If marked individuals (or identified individuals) are part of this project.	Character. Potential values: Yes, No
project_blank_images	If blank images were removed from this dataset.	Character. Potential values: Yes, Some, No
project_sensor_cluster	If sensors setup in pairs or clusters (groups of sensors).	Character. Potential values: Yes, No
project_admin	The name of the project administrator.	Character

project_admin_email	The email of the project administrator.	Character
project_admin_organization	The parent organization of the project.	Character
country_code	The country where the project is located, reported as a 3-letter ISO code.	Character. Potential values: Three-letter ISO codes are available at https://en.wikipedia.org/wiki/ISO_3166-1_alpha-3.
embargo	Length of embargo in months.	Integer. Range: 0-24
initiative_id	Unique ID generated by Wildlife Insights for an initiative.	Integer
metadata_license	The license assigned to the project metadata. Metadata includes all the information in this data dictionary along with any derived products that come from it.	Character. Potential values: CC0, CC-BY
image_license	The license assigned to the project media (i.e., images).	Character. Potential values: CC0, CC-BY, CC-BY-NC
data_citation	A suggested citation for the project that includes the authors, an ARK link to the project page and the date the project was last updated.	Character
count_optional	Indicates whether the data provider recorded count of objects in an image. The count is recorded in the images.csv as number_of_objects. If True, the count field was made optional in the project. If False, the count field was recorded.	Character. Potential values: True, False
project_type	Defines whether identifications within the project occurred at the image or sequence level.	Character. Potential values: Image, Sequence

Table 6. Variable information for the Snapshot_Europe_2021_2022_sequences.csv file, which contains information on when and during which deployment a sequence was captured, and the assigned classification (e.g. either 'blank', or details on the species captured).

**Variable identity**	**Variable definition**	**Data type**
project_id	An internal identification for the project.	Integer. Potential values: 2003388, 200446

deployment_id	Unique identifier for the camera deployment (within the project).	Character
sequence_id	Unique identifier for the sequence (within the project) generated by Wildlife Insights.	Integer.
is_blank	Is the image blank? Yes=1; No=0.	Integer. Potential values: 0, 1
identified_by	Name of the person who identified the photo. This will be marked as Computer Vision if the image has not been reviewed by project staff but has been identified by the AI model.	Character
wi_taxon_id	A permanent ID that is inherent to the Wildlife Insights Global Taxonomy.	Character. Reference WI Global Taxonomy table, available here: https://github.com/ConservationInternational/Wildlife-Insights----Data-Migration/tree/master/WI_Global_Taxonomy or in this table https://wildlifeinsights-taxonomy-ewutmovdfa-uc.a.run.app/
class	Scientific name for a Class.	Character
order	Scientific name for Order.	Character
family	Scientific name for Family.	Character
genus	Scientific name for a Genus in the image.	Character
species	Scientific name for a species in the image.	Character
common_name	Common name for a species in the image. Any identifications that are not wildlife (ex. cars, bycycles, etc) and types of humans (park staff, hunter,etc) are listed here.	Character

uncertainty	The uncertainty for the image identification by the photo identifier.	Character. Potential values: Absolutely sure, Pretty sure, Not sure, Don't know, Other. Missing value codes: NA
start_time	Date and time of the first image in a sequence.	Datetime. Format: YYYY-MM-DD hh:mm:ss
end_time	The date and time of the last image within a sequence.	Datetime. Format: YYYY-MM-DD hh:mm:ss
group_size	Number of animals per species in the sequence. Note: if a sequence has multiple species or individuals, each species or individual will require a separate record.	Integer.
age	Age of animal.	Character. Potential values: Adult, Juvenile, Unknown, Mixed. Missing value codes: NA
sex	Sex of animal.	Character. Potential values: Male, Female, Unknown, Mixed. Missing value codes: NA
animal_recognizable	Is the animal individually recognizable?	Logic. Potential values: True, False. Missing value codes: NA
individual_id	The individual animal ID.	Character. Missing value codes: NA
individual_animal_notes	Notes about the animal and/or the image itself.	Character. Missing value codes: NA
behavior	Notes about the animal and/or the image itself. Note that this maps to the field 'Remarks' in the User Interface.	Character. Missing value codes: NA
highlighted	Notes about the behavior of the animal.	Logic. True, False
markings	Distinct markings or dominant color of the animal.	Character. Missing value codes: NA
cv_confidence	The computer vision model's confidence in the prediction	Integer. Range: 0-100 (%). Missing value codes: NA

license	License assigned to the image. Any reuse of the image must follow the license terms.	Character. Potential values: CC0, CC-BY-4.0, CC-BY-NC

Table 7. Variable information for the Snapshot_Europe_2021_2022_images.csv file, which contains details on the image recorded (e.g. filename, storage location, etc.).

**Variable identity**	**Variable definition**	**Data type**
year	Identifier for specific Snapshot Europe survey, either 2021 or 2022	Integer. Potential values: 2021, 2022
project_id	An internal identification for the project.	Integer. Potential values: 2003388, 2004446
deployment_id	Unique identifier for the camera deployment (within the project).	Character
image_id	Unique identifier for the image (within the project) generated by Wildlife Insights.	Character
filename	The original filename of the image.	Character
sequence_id	Unique identifier for the sequence (within the project) generated by Wildlife Insights.	Integer
wi_taxon_id	A permanent ID that is inherent to the Wildlife Insights Global Taxonomy.	Character. Reference WI Global Taxonomy table, available here: https://github.com/ConservationInternational/Wildlife-Insights----Data-Migration/tree/master/WI_Global_Taxonomy or in this table https://wildlifeinsights-taxonomy-ewutmovdfa-uc.a.run.app/
class	Scientific name for a Class.	Character
order	Scientific name for Order.	Character
family	Scientific name for Family.	Character
genus	Scientific name for a Genus in the image.	Character
species	Scientific name for a species in the image.	Character
common_name	Common name for a species in the image. Any identifications that are not wildlife (ex. cars, bicycles, etc) and types of humans (park staff, hunter, etc) are listed here.	Character

timestamp	Date and time the image was taken	Datetime. Format: YYYY-MM-DD hh:mm:ss
bounding_boxes	Relative coordinates of one or more bounding boxes in the image. It will be in the format "{\"{\\\"detectionBox\\\": [ymin, xmin, ymax, xmax]}\"}". For example if the bounding box is this "{\"{\\\"detectionBox\\\": [0.421915054,0.63586086,0.647126615,0.780591547]}\"}" and the image size is 1080 pixels high x 1920 pixels wide, then the actual coordinates of the bounding box are ymin = 455.7 pixels, xmin = 1220.8 pixels, ymax = 698.9 pixels and xmax = 1498.7 pixels. If there are several bounding boxes in an image, there will be several "{\"{\\\"detectionBox\\\[coordinates]}\"}" separated by commas. The text is formated as JSON.	Character. Format: "{\"{\\\"detectionBox\\\":[ymin, xmin, ymax, xmax]}\"}"

Class V. Supplemental descriptors

A. Data acquisition

1. Data forms or acquisition methods: See Class II sections B2 and B3.

2. Location of completed data forms: NA

3. Data entry verification procedures: See Class III section A4 “Data verification”

B. Quality assurance/quality control procedures: See Class III section A4 “Data verification”

C. Related materials: NA

D. Computer programs and data-processing algorithms: None

E. Archiving

1. Archival procedures: Data are archived in Edmond – the Open Research Data Repository of the Max Planck Society: https://doi.org/10.17617/3.7YLWMU, for long-term storage.

2. Redundant archival sites: A copy of the data is archived locally in the servers of the Max Planck Institute of Animal Behavior, Konstanz, Germany. Data from each year are also stored in Wildlife Insights (projects: Snapshot Europe 2021, Iannarilli et al. 2025a, and Snapshot Europe 2022, Iannarilli et al. 2025b, respectively); however, we recommend to rather use the data associated to this publication as it has undergone further data cleaning.

F. Publications and results: None

G. History of data set usage

1. Data request history: None

2. Data set update history: None

3. Review history: None

4. Questions and comments from secondary users: None

ACKNOWLEDGMENTS

The collection of this dataset was only possible thanks to the volunteer work and effort of all the people and institutions involved. The Max Planck Institute of Animal Behavior provided support for the management and coordination of the initiative. We are grateful to Carla Avolio and Imran Razik for their help with communication and social media. Team-specific acknowledgments follow:
AT_Forest_National Park Hohe Tauern: Hohe Tauern National Park Salzburg; AT_Forest_Wilderness Area Dürrenstein: The administration of the Wilderness Area Dürrenstein-Lassingtal and the Forestry Administration Langau for their support and permission for the fieldwork; BE_Wetland_Leuven: All the vollunteers and the colleagues of the insitute that participated during the fieldwork; CZ_Forest_PLA Broumovsko: Jan Koranda and volunteers of Carnivore Tracking Project; DE_Agricultural_Bettenreute: Daniel Burchard and the interns within the project for assisting in data collection; DE_Agricultural_District of Goettingen: Robin Doerr; DE_Forest_Hörre: Everyone who contributed to the planning, implementation, and image classification – especially Teresa Döngens, Mona Hutter, and HessenForst with the Wetzlar Forest Office; DE_Forest_Krofdorf: Holger Brusius and the staff at Forstamt Wettenberg for support with field work; DE_Forest_Langenau: Academy for the Protection of Zoo Animals and Wildlife e.V; DE_Forest_Schwarzwald: ForstBW for the collaboration and providing the research plots; DE_Forest_Solling: Christine Huning; DE_Wetland_Gut Klepelshagen: Michael Tetzlaff for help collecting data; ES_Forest_Collsacabra: Several volunteers that helped to deploy and revise cameras: Xavier Arumí, Alfred Encuentra, Jordi Lladó, Blanca Monteis, Pau Pérez, Eric Serratosa, Marc Rota, Joel Vidal and David Vilalta; Grup de Naturalistes d'Osona (GNO-ICHN), Institució Catalana d'Història Natural (ICHN) for support; ES_ShrubbyVeg_Parque Nacional de Doñana; ES_ShrubbyVeg_Parque Nacional de Doñana B: Fundación Biodiversidad, Ministerio para la Transición Ecológica y el Reto Demográfico, Project AI-CENSUS, Programa Operativo FEDER Andalucía 2014–2020, Project UHU-202028; ES_Wetland_Aiguamolls Empordà: Laura Adan (undergraduate student) and Parc Natural dels Aiguamolls de l'Empordà. Generalitat de Catalunya; GB_Agricultural_Carlton Marshes; GB_Agricultural_Foxburrow Farm: Austin Tolton, Borislava Gacheva, Fletcher Telling, Charlotte Beck, Students on the Wildife, Ecology and Conservation Science Degree at the University of Suffolk, Patrick Barker and the staff at Lodge Farm, Westhorpe, Lewis Yates, JJ Ratcliff, Ben Calvesbert, and the staff and volunteers of the Suffolk Wildlife Trusts; GB_Agricultural_Holywood: National Museums NI for allowing the work to take place at the Ulster Folk Museum. ; GB_Agricultural_RVC Farm: Graeme Webster, Boltons Park Farm, Royal Veterinary College; GB_Agricultural_Yorkshire: The research assistants and students from the British Carnivore Project; IE_Forest_County Wicklow: Danny Alvey of ReWild Wicklow, ReWild Wicklow volunteers and donors, University College Dublin School of Biology and Environmental Science staff and students, Coillte - especially Barry Coad, the

National Parks and Wildlife Service - especially Hugh McLindon, the Local Authority Waters Programme, and all private landowners who hosted camera traps; GR_Forest_Nimfeo: Sampling in Greece by the ARCTUROS team was carried out within the framework of the project “Large Carnivore Connect: targeted conservation actions for maintaining/promoting large carnivore connectivity in the southwestern Balkans”, supported by the Prespa Ohrid Nature Trust. PONT support for the production of this publication does not constitute an endorsement of the contents, which reflect the views only of the authors, and PONT cannot be held responsible for any use which may be made of the information contained therein; IT_Forest_Castelporziano: Servizio Tenuta Presidenziale di Castelporziano and the Settore Tutela e Gestione Ambientale for logistics support to field activities; IT_Forest_Maxcine: Fabio Cianchi and WWF Oasi; IT_Forest_Parco Nazionale Casentino e IT_Forest_Parco Nazionale Casentino: The staff of the Foreste Casentinesi National Park and the Reparto Carabinieri Parco Foreste Casentinesi and Reparto Carabinieri Biodiversità di Pratovecchio for their help with data collection; Matilde Marconi and Giulia Cannoni for their help with the identification of camera-trap images; IT_Forest_Parco Regionale Paneveggio: All the personnel of Parco Naturale Paneveggio Pale di San Martino, particularly Piergiovanni Partel, Enrico Dorigatti, Gilberto Volcan and Alessandro Forti, for invaluable support during fieldwork and sampling design; IT_Forest_Salbertrand: Ente di Gestione dei Parchi delle Alpi Cozie; LT_Forest_Labanoras: For assistance with equipment and deployment Viktoras Mažuolis, Danas Mažuolis, Tomas Rimkus, Rūta Pašukonytė; MK_Forest_Mavrovo National Park North, MK_Forest_Mavrovo National Park North: Mavrovo National Park; NO_Forest_Evenstad: Research group WildCom; PL_Forest_Celestynów District North, PL_Forest_Celestynów District South: The authorities of the Celestynów Forest District for permission to conduct research within the district; PT_ArtNonAg_Montado Grandola; PT_ArtNonAg_Montado Companhia Lezirias: Diogo Raposo and João Almeida; RO_Forest_Putna-Vrancea Natural Park: Andrei Dinu, Mihai Pop, Matei Dragomir, Cosmin Taru, Andreea Taru, Steluta Manolache, Ilenia Marocco, Laurentiu Rozylowicz ; RO_Forest_Rezervatia Naturala Padurea Merisor-Cotul Zatuanului; Cosmin Taru, Andreea Taru, Andrei Dinu, Ilenia Marocco; SE_Forest_Södermanland East, SE_Forest_Södermanland West: The forest owners that allowed us to set up the camera traps in their forest; SE_Forest_Svartberget, SE_Forest_Svartberget, SE_Forest_Grimsö: Emilia Arkdalen, Nadine Erath, Gunnar Jansson, Daniel Jung, Pablo Palencia, Pablo del Rio and the Swedish Infrastructure for Ecosystem Science (SITES) at Grimsö Wildlife Research Station; TR_Forest_Sarikamis: The staff and volunteers of the KuzeyDoga Society for their help, Batubay Özkan and Barbara Watkins for their generous support through the years, the Fondation Segré and the Sigrid Rausing Trust for providing the majority of the funding for this project, and other generous donors, including Bilge Bahar, Faruk Eczacıbaşı, Seha İşmen, Ömer Koç, Ömer Külahçıoğlu, Burak Över, Batubay Özkan, Emin Özgür, Suna Reyent, Ceren Sağlamer, Barbara Watkins, and the Whitley Fund.

Literature Citations

Burton, A. C., C. Beirne, K. M. Gaynor, C. Sun, A. Granados, M. L. Allen, J. M. Alston, G. C. Alvarenga, F. S. Á. Calderón, Z. Amir, C. Anhalt-Depies, C. Appel, S. Arroyo-Arce, G. Balme, A. Bar-Massada, D. Barcelos, E. Barr, E. L. Barthelmess, C. Baruzzi, S. M. Basak, N. Beenaerts, J. Belmaker, O. Belova, B. Bezarević, T. Bird, D. A. Bogan, N. Bogdanović, A. Boyce, M. Boyce, L. Brandt, J. F. Brodie, J. Brooke, J. W. Bubnicki, F. Cagnacci, B. S. Carr, J. Carvalho, J. Casaer, R. Černe, R. Chen, E. Chow, M. Churski, C. Cincotta, D. Ćirović, T. D. Coates, J. Compton, C. Coon, M. V. Cove, A. P. Crupi, S. D. Farra, A. K. Darracq, M. Davis, K. Dawe, V. De Waele, E. Descalzo, T. A. Diserens, J. Drimaj, M. Duľa, S. Ellis-Felege, C. Ellison, A. Ertürk, J. Fantle-Lepczyk, J. Favreau, M. Fennell, P. Ferreras, F. Ferretti, C. Fiderer, L. Finnegan, J. T. Fisher, M. C. Fisher-Reid, E. A. Flaherty, U. Fležar, J. Flousek, J. M. Foca, A. Ford, B. Franzetti, S. Frey, S. Fritts, Š. Frýbová, B. Furnas, B. Gerber, H. M. Geyle, D. G. Giménez, A. J. Giordano, T. Gomercic, M. E. Gompper, D. M. Gräbin, M. Gray, A. Green, R. Hagen, R. B. Hagen, S. Hammerich, C. Hanekom, C. Hansen, S. Hasstedt, M. Hebblewhite, M. Heurich, T. R. Hofmeester, T. Hubbard, D. Jachowski, P. A. Jansen, K. J. Jaspers, A. Jensen, M. Jordan, M. C. Kaizer, M. J. Kelly, M. T. Kohl, S. Kramer-Schadt, M. Krofel, A. Krug, K. M. Kuhn, D. P. J. Kuijper, E. K. Kuprewicz, J. Kusak, M. Kutal, D. J. R. Lafferty, S. LaRose, M. Lashley, R. Lathrop, T. E. Lee Jr, C. Lepczyk, D. B. Lesmeister, A. Licoppe, M. Linnell, J. Loch, R. Long, R. C. Lonsinger, J. Louvrier, M. S. Luskin, P. MacKay, S. Maher, B. Manet, G. K. H. Mann, A. J. Marshall, D. Mason, Z. McDonald, T. McKay, W. J. McShea, M. Mechler, C. Miaud, J. J. Millspaugh, C. M. Monteza-Moreno, D. Moreira-Arce, K. Mullen, C. Nagy, R. Naidoo, I. Namir, C. Nelson, B. O'Neill, M. T. O'Mara, V. Oberosler, C. Osorio, F. Ossi, P. Palencia, K. Pearson, L.

Pedrotti, C. E. Pekins, M. Pendergast, F. F. Pinho, R. Plhal, X. Pocasangre-Orellana, M. Price, M. Procko, M. D. Proctor, E. E. Ramalho, N. Ranc, S. Reljic, K. Remine, M. Rentz, R. Revord, R. Reyna-Hurtado, D. Risch, E. G. Ritchie, A. Romero, C. Rota, F. Rovero, H. Rowe, C. Rutz, M. Salvatori, D. Sandow, C. M. Schalk, J. Scherger, J. Schipper, D. G. Scognamillo, Ç. H. Şekercioğlu, P. Semenzato, J. Sevin, H. Shamon, C. Shier, E. A. Silva-Rodríguez, M. Sindicic, L. K. Smyth, A. Soyumert, T. Sprague, C. C. St Clair, J. Stenglein, P. A. Stephens, K. M. Stępniak, M. Stevens, C. Stevenson, B. Ternyik, I. Thomson, R. T. Torres, J. Tremblay, T. Urrutia, J.-P. Vacher, D. Visscher, S. L. Webb, J. Weber, K. C. B. Weiss, L. S. Whipple, C. A. Whittier, J. Whittington, I. Wierzbowska, M. Wikelski, J. Williamson, C. C. Wilmers, T. Windle, H. U. Wittmer, Y. Zharikov, A. Zorn, and R. Kays. 2024. Mammal responses to global changes in human activity vary by trophic group and landscape. Nature ecology & evolution.

Cove, M. V., R. Kays, H. Bontrager, C. Bresnan, M. Lasky, T. Frerichs, R. Klann, T. E. Lee Jr, S. C. Crockett, A. P. Crupi, K. C. B. Weiss, H. Rowe, T. Sprague, J. Schipper, C. Tellez, C. A. Lepczyk, J. E. Fantle-Lepczyk, S. LaPoint, J. Williamson, M. C. Fisher-Reid, S. M. King, A. J. Bebko, P. Chrysafis, A. J. Jensen, D. S. Jachowski, J. Sands, K. A. MacCombie, D. J. Herrera, M. van der Merwe, T. W. Knowles, R. V. Horan 3rd, M. S. Rentz, L. S. E. Brandt, C. Nagy, B. T. Barton, W. C. Thompson, S. P. Maher, A. K. Darracq, G. Hess, A. W. Parsons, B. Wells, G. W. Roemer, C. J. Hernandez, M. E. Gompper, S. L. Webb, J. P. Vanek, D. J. R. Lafferty, A. M. Bergquist, T. Hubbard, T. Forrester, D. Clark, C. Cincotta, J. Favreau, A. N. Facka, M. Halbur, S. Hammerich, M. Gray, C. C. Rega-Brodsky, C. Durbin, E. A. Flaherty, J. M. Brooke, S. S. Coster, R. G. Lathrop, K. Russell, D. A. Bogan, R. Cliché, H. Shamon, M. T. R. Hawkins, S. B. Marks, R. C. Lonsinger, M. T. O'Mara, J. A. Compton, M. Fowler, E. L. Barthelmess, K. E. Andy, J. L. Belant, D. E. Beyer Jr, T. M. Kautz, D. G. Scognamillo, C. M. Schalk,

M. S. Leslie, S. L. Nasrallah, C. N. Ellison, C. Ruthven, S. Fritts, J. Tleimat, M. Gay, C. A. Whittier, S. A. Neiswenter, R. Pelletier, B. A. DeGregorio, E. K. Kuprewicz, M. L. Davis, A. Dykstra, D. S. Mason, C. Baruzzi, M. A. Lashley, D. R. Risch, M. R. Price, M. L. Allen, L. S. Whipple, J. H. Sperry, R. H. Hagen, A. Mortelliti, B. E. Evans, C. E. Studds, A. P. K. Sirén, J. Kilborn, C. Sutherland, P. Warren, T. Fuller, N. C. Harris, N. H. Carter, E. Trout, M. Zimova, S. T. Giery, F. Iannarilli, S. D. Higdon, R. S. Revord, C. P. Hansen, J. J. Millspaugh, A. Zorn, J. F. Benson, N. H. Wehr, J. N. Solberg, B. D. Gerber, J. C. Burr, J. Sevin, A. M. Green, Ç. H. Şekercioğlu, M. Pendergast, K. A. Barnick, A. J. Edelman, J. R. Wasdin, A. Romero, B. J. O'Neill, N. Schmitz, J. M. Alston, K. M. Kuhn, D. B. Lesmeister, M. A. Linnell, C. L. Appel, C. Rota, J. L. Stenglein, C. Anhalt-Depies, C. Nelson, R. A. Long, K. Jo Jaspers, K. R. Remine, M. J. Jordan, D. Davis, H. Hernández-Yáñez, J. Y. Zhao, and W. J. McShea. 2021. SNAPSHOT USA 2019: a coordinated national camera trap survey of the United States. Ecology 102:e03353.

Doser, J. W., A. O. Finley, M. Kéry, and E. F. Zipkin. 2022. spOccupancy: An R package for single-species, multi-species, and integrated spatial occupancy models. Methods in ecology and evolution / British Ecological Society 13:1670–1678.

European Environmental Agency. 2016. EU Biogeographical Regions.

Fukasawa, K., T. Morosawa, Y. Nakashima, S. Takagi, T. Yokoyama, M. Ando, H. Iijima, M. U. Saito, N. Kumada, K. Tochigi, A. Yoshioka, S. Funatsu, S. Koike, H. Uno, T. Enomoto, W. McShea, and R. Kays. 2025. Snapshot Japan 2023: the first camera trap dataset under a globally standardised protocol in Japan. Biodiversity data journal 13:e141168.

Gadot, T., Ștefan Istrate, H. Kim, D. Morris, S. Beery, T. Birch, and J. Ahumada. 2024. To crop or not to crop: Comparing whole-image and cropped classification on a large

dataset of camera trap images. IET computer vision.

Iannarilli, F., B. Keeves-von Wolff, M. Wikelski, M. Heurich, R. Kays, and F. Cagnacci. 2025a, February. Snapshot Europe 2021. Accessed via wildlifeinsights.org on 2025-03-17. http://n2t.net/ark:/63614/w12003388

Iannarilli, F., B. Keeves-von Wolff, M. Wikelski, M. Heurich, R. Kays, and F. Cagnacci. 2025b, February. Snapshot Europe 2022. Accessed via wildlifeinsights.org on 2025-03-17. http://n2t.net/ark:/63614/w12004446

Kays, R., M. V. Cove, J. Diaz, K. Todd, C. Bresnan, M. Snider, T. E. Lee Jr, J. G. Jasper, B. Douglas, A. P. Crupi, K. C. B. Weiss, H. Rowe, T. Sprague, J. Schipper, C. A. Lepczyk, J. E. Fantle-Lepczyk, J. Davenport, M. Zimova, Z. Farris, J. Williamson, M. C. Fisher-Reid, D. Rezendes, S. M. King, P. Chrysafis, A. J. Jensen, D. S. Jachowski, K. C. King, D. J. Herrera, S. Moore, M. van der Merwe, J. V. Lombardi, M. Sergeyev, M. E. Tewes, R. V. Horan 3rd, M. S. Rentz, A. Driver, L. R. S. E. Brandt, C. Nagy, P. Alexander, S. P. Maher, A. K. Darracq, E. G. Barr, G. Hess, S. L. Webb, M. D. Proctor, J. P. Vanek, D. J. R. Lafferty, T. Hubbard, J. E. Jiménez, C. McCain, J. Favreau, J. Fogarty, J. Hill, S. Hammerich, M. Gray, C. C. Rega-Brodsky, C. Durbin, E. A. Flaherty, J. Brooke, S. S. Coster, R. G. Lathrop, K. Russell, D. A. Bogan, H. Shamon, B. Rooney, A. Rockhill, R. C. Lonsinger, M. T. O'Mara, J. A. Compton, E. L. Barthelmess, K. E. Andy, J. L. Belant, T. Petroelje, N. H. Wehr, D. E. Beyer Jr, D. G. Scognamillo, C. Schalk, K. Day, C. N. Ellison, C. Ruthven, B. Nunley, S. Fritts, C. A. Whittier, S. A. Neiswenter, R. Pelletier, B. A. DeGregorio, E. K. Kuprewicz, M. L. Davis, C. Baruzzi, M. A. Lashley, B. McDonald, D. Mason, D. R. Risch, M. L. Allen, L. S. Whipple, J. H. Sperry, E. Alexander, P. J. Wolff, R. H. Hagen, A. Mortelliti, A. Bolinjcar, A. M. Wilson, S. Van Norman, C. Powell, H. Coletto, M. Schauss, H. Bontrager, J. Beasley, S. N. Ellis-Felege, S. R. Wehr, S. T. Giery, C. E. Pekins, S. H. LaRose, R. S. Revord, C. P. Hansen,

L. Hansen, J. J. Millspaugh, A. Zorn, B. D. Gerber, K. Rezendes, J. Adley, J. Sevin, A. M. Green, Ç. H. Şekercioğlu, M. E. Pendergast, K. Mullen, T. Bird, A. J. Edelman, A. Romero, B. J. O'Neill, N. Schmitz, R. A. Vandermus, J. M. Alston, K. M. Kuhn, S. C. Hasstedt, D. B. Lesmeister, C. L. Appel, C. Rota, J. L. Stenglein, C. Anhalt-Depies, C. L. Nelson, R. A. Long, K. R. Remine, M. J. Jordan, L. M. Elbroch, D. Bergman, S. Cendejas-Zarelli, K. Sager-Fradkin, M. Conner, G. Morris, E. Parsons, H. Hernández-Yáñez, and W. J. McShea. 2022. SNAPSHOT USA 2020: A second coordinated national camera trap survey of the United States during the COVID-19 pandemic. Ecology 103:e3775.

Kays, R., & Parsons, A. W. (2014). Mammals in and around suburban yards, and the attraction of chicken coops. Urban Ecosystems, 17, 691–705. https://doi.org/10.1007/s11252-014-0347-2

Mackenzie, D. I., J. D. Nichols, G. B. Lachman, S. Droege, J. Andrew, C. a. Langtimm, S. Ecology, and N. Aug. 2002. Estimating site occupancy rates when detection probabilities are less than one. Ecology 83:2248–2255.

R Core Team. 2022. R: A language and environment for statistical computing. R Foundation for Statistical Computing, Vienna, Austria.

Parsons, A. W., Bland, C., Forrester, T., Baker-Whatton, M. C., Schuttler, S. G., Mcshea, W. J., Costello, R., & Kays, R. (2016). The ecological impact of humans and dogs on wildlife in protected areas in eastern North America. Biological Conservation, 203, 75–88. https://doi.org/10.1016/j.biocon.2016.09.001

Rooney, B., R. Kays, M. V. Cove, A. Jensen, B. R. Goldstein, C. Pate, P. Castiblanco, M. E. Abell, J. Adley, B. Agenbroad, A. A. Ahlers, P. D. Alexander, D. Allen, M. L. Allen, J. M. Alston, M. Alyetama, T. L. Anderson, R. Andrade, C. Anhalt-Depies, C. L. Appel, L. Armendariz, C. R. Ayers, A. B. Baird, C. Bak, G. Bandler, E. E. Barding, E. G. Barr,

C. Baruzzi, K. Bashaw, S. C. Beers, J. L. Belant, E. Bell, J. F. Benson, A. Berg, D. L. Bergman, B. M. Bernhardt, M. A. Bethel, T. Bird, A. B. Bishop, D. A. Bogan, L. Brandt, L. C. Brandt, A. B. Branney, C. Bratton, C. E. Bresnan, J. M. Brooke, E. K. Buchholtz, F. Buderman, A. D. Burnett, E. E. Burns, D. A. Byrd, S. A. Cannella, K. A. Carey, W. A. Carlile, K. L. Carter, B. J. Cassidy, I. Castro-Arellano, S. Cendejas-Zarelli, N. Chatterjee, A. E. Cheeseman, C. Chevalier, M. C. Chitwood, P. Chrysafis, B. A. Collier, D. P. Collins, J. A. Compton, R. Cone, L. M. Conner, B. L. Cook, O. G. Cosby, S. S. Coster, A. P. Crupi, A. K. Darracq, J. M. Davenport, D. Davis, D. R. Davis, M. L. Davis, R. J. Davis, B. A. DeGregorio, A. Deshwal, K. D. Dougherty, A. Drauglis, C. J. Durbin, A. J. Edelman, V. Elder, B. Eller, E. H. Ellington, S. N. Ellis-Felege, C. N. Ellison, J. E. Fantle-Lepczyk, J. J. Farr, Z. J. Farris, S. P. Finnegan, M. C. Fisher-Reid, E. A. Flaherty, G. Franzoi Dri, S. Fritts, J. Fuller, T. Gallo, L. S. Ganoe, C. N. Ganong, R. Garibay, B. D. Gerber, F. D. Gerraty, S. T. Giery, S. M. Gilyot, J. L. Glasscock, B. Goldfarb, L. E. Good, G. Granados, A. M. Green, J. K. Grewal, A. Grusenmeyer, J. M. Guthrie, M. T. Hallett, C. Hansen, L. P. Hansen, C. Hanson, E. J. Harrity, S. C. M. Hasstedt, M. Hebblewhite, D. J. Herrera, A. Holland, B. R. Humphreys, H. D. Island, A. R. Jack, E. P. Johansson, A. M. Johnson, L. Johnson, T. L. Johnstone-Yellin, M. L. S. P. Jorge, W. Kahano, M. A. Kinsey, B. E. Klossing, T. W. Knowles, M. M. Koeck, J. L. Koprowski, K. M. Kuhn, E. K. Kuprewicz, D. J. R. Lafferty, J. A. Lamberton-Moreno, T. J. Land, A. M. Langston, S. LaPoint, E. N. Largent, M. A. Lashley, R. G. Lathrop, T. E. Lee Jr, C. A. Lepczyk, D. B. Lesmeister, C. Leung, J. V. Lombardi, R. Long, R. C. Lonsinger, I. Lord, S. S. Madere, S. P. Maher, J. A. Mallinoff, A. Martinez, D. S. Mason, H. A. Mathewson, A. E. Mayer, K. P. McCarthy, S. F. McCracken, B. McDonald, B. McGarry, S. T. McMurry, L. E. McTigue, B. M. Mena, M. Mercer, M. R. Merz, S. Millar, G. D. Miller, J. J. Millspaugh, R. J. Moll, T. W. Mong, J. D. Monzón, J.

C. Moore, A. Mortelliti, K. W. Mote, K. Mullen, A. Mychajliw, C. Nagy, S. A. Neiswenter, D. R. Neyland, L. P. Nicholson, M. T. O'Mara, B. J. O'Neill, E. A. Olson, M. J. Orgill, G. Palomo-Munoz, S. M. Parsons, L. E. Patrick, J. R. Patterson, D. L. Pearce, M. E. Pendergast, B. S. Perla, T. R. Petroelje, H. Pliske, M. K. P. Poisson, M. R. Price, M. D. Proctor, N. J. Proudman, J. L. Rachlow, R. E. Ramos, M. Reabold, J. Redinger, A. E. Reed, C. C. Rega-Brodsky, E. Rehm, K. R. Remine, M. S. Rentz, E. Ridder, D. R. Risch, L. L. Robbins, J. P. Roemer, A. Romero, C. Rota, C. M. Schalk, B. D. Scholten, C. L. Scott, B. M. Scurlock, M. Sergeyev, W. J. Severud, J. Sevin, H. Shamon, C. Sharp, M. Shaw, V. Siverls-Dunham, A. B. Smith, D. S. Smith, M. H. Snider, D. A. Sossover, A. R. Sovie, J. A. Sparks, J. Speiser, M. T. Springer, J. L. Spurlin, E. A. Steinkamp, J. L. Stenglein, J. Stewart Kloker, C. M. Stitzman, M. Stokes, K. Stringer, J. Stutzman, D. S. Sullins, C. Sullivan, N. B. Sullivan, E. P. Tanner, A. M. Tanner, E. B. Thornock, J. Titus, J. M. Tleimat, K. Toomey, L. T. Toussaint, M. Uribe, M. Van der Merwe, D. J. Van Parys, J. P. Vanek, J. Varner, B. V. Walker, C. Wallace, D. Ward, B. H. Warner, D. T. Warren, J. R. Wasdin, S. L. Webb, K. L. Wehr, N. H. Wehr, E. G. Weigel, T. J. Werdel, L. S. Whipple, C. A. Whittier, C. Wiersema, A. M. Wilson, M. F. H. Wilson, A. J. Wolf, J. P. Wolford, D. W. Wolfson, D. J. Woolsey, M. A. Wuensch, G. Xu, K. L. Yurewicz, V. Zancho, M. Zimova, A. Zorn, and W. J. McShea. 2025. SNAPSHOT USA 2019–2023: The first five years of data from a coordinated camera trap survey of the United States. Global ecology and biogeography: a journal of macroecology 34:e13941.

Shamon, H., R. Maor, M. V. Cove, R. Kays, J. Adley, P. D. Alexander, D. N. Allen, M. L. Allen, C. L. Appel, E. Barr, E. L. Barthelmess, C. Baruzzi, K. Bashaw, G. Bastille-Rousseau, M. E. Baugh, J. Belant, J. F. Benson, B. A. Bespoyasny, T. Bird, D. A. Bogan, L. S. E. Brandt, C. E. Bresnan, J. M. Brooke, F. E. Buderman, S. G. Buzzell, A.

E. Cheeseman, M. C. Chitwood, P. Chrysafis, M. K. Collins, D. P. Collins, J. A. Compton, L. M. Conner, O. G. Cosby, S. S. Coster, B. Crawford, A. P. Crupi, A. K. Darracq, M. L. Davis, B. A. DeGregorio, K. L. Denningmann, K. D. Dougherty, A. Driver, A. J. Edelman, E. H. Ellington, S. N. Ellis-Felege, C. N. Ellison, J. E. Fantle-Lepczyk, Z. J. Farris, J. Favreau, P. Fernandez, M. C. Fisher-Reid, M. C. Fitzpatrick, E. A. Flaherty, T. D. Forrester, S. R. Fritts, T. Gallo, B. D. Gerber, S. T. Giery, J. L. Glasscock, A. D. Gonatas, A. C. Grady, A. M. Green, T. Gregory, N. Griffin, R. H. Hagen, C. P. Hansen, L. P. Hansen, S. C. Hasstedt, H. Hernández-Yáñez, D. J. Herrera, R. V. Horan 3rd, V. L. Jackson, L. Johnson, M. J. Jordan, W. Kahano, J. Kiser, T. W. Knowles, M. M. Koeck, C. Koroly, K. M. Kuhn, E. K. Kuprewicz, D. J. R. Lafferty, S. D. LaPoint, M. Lashley, R. G. Lathrop, T. E. Lee Jr, C. A. Lepczyk, D. B. Lesmeister, J. V. Lombardi, R. A. Long, R. C. Lonsinger, P. MacKay, S. P. Maher, D. S. Mason, J. J. Millspaugh, R. J. Moll, J. B. Moon, A. Mortelliti, A. M. Mychajliw, C. M. Nagy, S. A. Neiswenter, D. L. Nelson, C. E. Nemes, C. K. Nielsen, E. Olson, M. T. O'Mara, B. J. O'Neill, B. R. Page, E. Parsons, B. S. Pease, M. E. Pendergast, M. Proctor, H. Quick, C. C. Rega-Brodsky, M. S. Rentz, K. Rezendes, D. Rich, D. R. Risch, A. Romero, B. R. Rooney, C. T. Rota, C. A. Samples, C. M. Schalk, Ç. H. Sekercioğlu, M. Sergeyev, A. B. Smith, D. S. Smith, J. H. Sperry, J. L. Stenglein, M. K. Stokes, J. S. Stutzman, K. R. Todd, J. P. Vanek, W. Varga, Z. M. Wardle, S. L. Webb, N. H. Wehr, L. S. Whipple, C. A. Whittier, J. S. Widness, J. Williamson, A. M. Wilson, A. J. Wolf, M. Zimova, A. S. Zorn, and W. J. McShea. 2024. SNAPSHOT USA 2021: A third coordinated national camera trap survey of the United States. Ecology 105:e4318.

Yasuda, M. (2004). Monitoring diversity and abundance of mammals with camera traps: a case study on Mount Tsukuba, central Japan. Mammal Study, 29, 37–46. https://www.jstage.jst.go.jp/article/mammalstudy/29/1/29_1_37/_pdf/-char/en
